# Learning in Interactive Decision-Making: The Interplay Between Cognitive Abilities and the Strategic Environment

**DOI:** 10.1162/opmi_a_00186

**Published:** 2025-01-23

**Authors:** Joshua Zonca, Lilia Del Mauro, Aldo Rustichini, Luca Polonio, Carlo Reverberi

**Affiliations:** Department of Psychology, University of Milano-Bicocca, Milan, Italy; Milan Center for Neuroscience - NeuroMI, University of Milano-Bicocca, Milan, Italy; Department of Economics, University of Minnesota, Minneapolis, MN, USA; Department of Economics, Management and Statistics, University of Milano-Bicocca, Milan, Italy

**Keywords:** learning, strategic sophistication, interactive decision-making, cognitive abilities, learning environment

## Abstract

A remarkable feature of human intelligence is the ability to optimize our decisions based on the potential actions of others. This ability, i.e., strategic sophistication, is crucial in strategic interactions, where we need to predict others’ actions (first-order beliefs), anticipate others’ beliefs about our own possible actions (second-order beliefs), and optimize decisions based on such beliefs. While behavioral research has highlighted systematic departures from theoretically optimal behavior in strategic interactions, little is known about the possibility of enhancing strategic sophistication. In particular, no studies investigated whether and how the interaction between exogenous factors (i.e., the learning environment) and endogenous factors (i.e., individual cognitive abilities) shapes learning in strategic settings. In a novel mouse-tracking study, we manipulate the learning environment and test its interaction with individual cognitive abilities in determining context-specific and transfer of learning in interactive games. Choice and process data reveal that the interplay between individual cognitive abilities and the learning environment does modulate participants’ learning. The learning environment determines *what* is learned and *whether* acquired knowledge is applied in similar contexts and transferred to novel settings. Moreover, learning success in different strategic environments depends on individual cognitive abilities. In particular, higher levels of cognitive reflection are necessary to learn sophisticated strategic behavior (i.e., forming second-order beliefs) and transfer acquired knowledge to more complex strategic environments after receiving relevant feedback. However, higher cognitive reflection levels are insufficient to prevent the misapplication of procedures learned in a specific environment to other strategic contexts with substantial structural differences. Our results provide novel insights into the factors that promote or hamper learning in interactive decision-making.

## INTRODUCTION

Imagine a car driver deciding the route to take in traffic, a company trying to reach a patent before its competitors, a nation in conflict against another. Despite the differences between these scenarios, they all share a critical feature: the outcome of one actor’s decisions is influenced by other actors’ decisions. We refer to these types of scenarios as strategic interactions. Game theory (Fudenberg & Tirole, 1991; Maschler et al., 2020) mathematically models strategic interactions, enabling real-life applications in numerous contexts including education (Burguillo, 2010), financial markets (Allen & Morris, 2014), politics (Brams, 2011; McKelvey & Patty, 2006), military and defense strategy (Maschler et al., 2020), safety management (Meng et al., 2020), job markets (Bangerter et al., 2012), and machine learning (Hazra & Anjaria, 2022).

**Figure 1.**
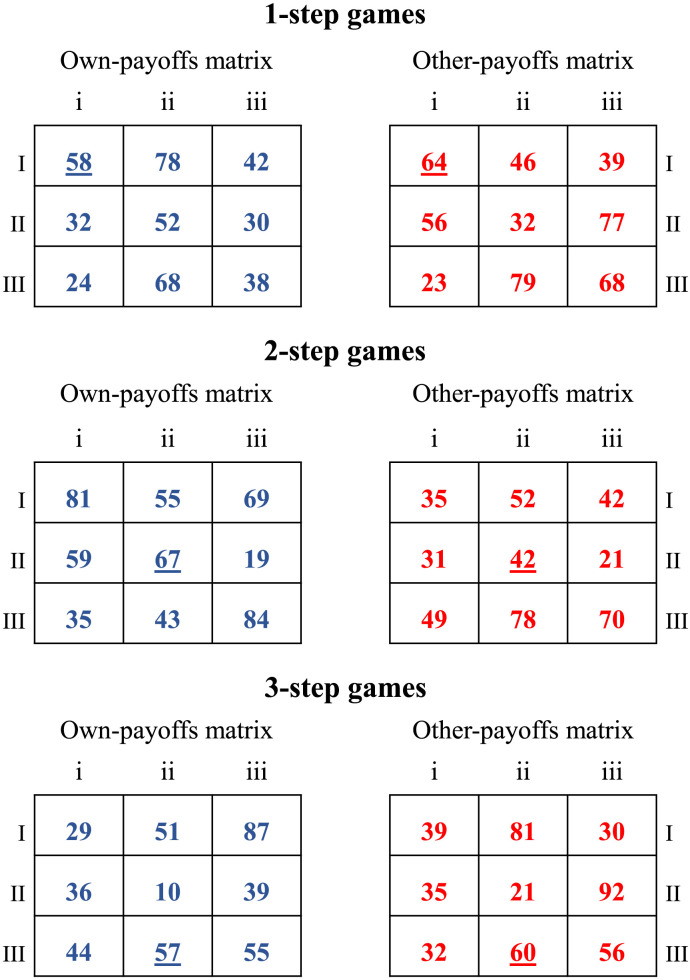
Examples of 1-step, 2-step, and 3-step games. The matrix on the left contains all the row player’s potential actions (I, II, III) and payoffs (in blue). The matrix on the right contains all the column player’s potential actions (i, ii, iii) and payoffs (in red). In the current experiment, participants covered the role of row players, whereas the computer played as a column player. The final outcome of the game depends on the combination of the actions of the two players. For instance, in the upper game (1-step games), if the row player selects row I, and the column player chooses column ii, the row and the column players gain 78 and 46 points, respectively. The underlined payoffs indicate the game’s Nash equilibrium solution.

In strategic interactions, optimal decisions rely on the ability to form accurate beliefs about others’ intentions (first-order beliefs), on others’ expectations about our potential actions (second-order beliefs), and, eventually, best respond to such beliefs. We refer to “strategic sophistication” as the ability to manage the more complex instances of such recursive reasoning about others and the self, i.e., multiple “steps” of strategic thinking. To investigate individuals’ strategic behavior in interactive settings, we can use interactive games. Whenever two players interact, the game’s strategic form can be represented by a payoff matrix, i.e., a table representing the outcomes of each possible combination of actions (see Figure 1). Extensive evidence from interactive games has revealed a remarkable heterogeneity of people’s strategic sophistication. In many strategic contexts, the observed behavior of players does not align with the assumptions of *classical* normative models of strategic behavior, such as full rationality and correct beliefs about others’ actions (Nash, 1950). Different theories have aimed to account for the observed behavioral heterogeneity in strategic choices by relaxing some of the assumptions of classical models. Non-equilibrium models of strategic thinking like level-k (Costa-Gomes & Crawford, 2006; Costa-Gomes et al., 2001; Crawford et al., 2013; Nagel, 1995; Stahl & Wilson, 1995) and Cognitive Hierarchy (CH, Camerer et al., 2004; Chong et al., 2016) relax the assumptions of full rationality and belief consistency, allowing players to have potentially incorrect beliefs about other’s rationality. In particular, these theories claim that players regard others as less sophisticated than themselves and respond consistently to this belief. A recent extension of the level-k approach (Alaoui & Penta, 2016) models behavioral heterogeneity by accounting for the interactions between depth of reasoning, players’ incentives, cognitive bounds, and cognitive costs. In particular, the model allows players to adapt their level of strategic sophistication based on beliefs about their counterparts’ strategic level, cognitive costs, and current incentives; however, individual cognitive bounds can limit adaptation to higher levels of strategic thinking. Another recent model (Frydman & Nunnari, 2021), introduced the concept of *cognitive imprecision* to explain strategic suboptimality: cognitive noise can distort the encoding and representation of matrix payoffs, leading to suboptimal choices. These recent theoretical approaches highlight the tension between 1) the possibility of modulating strategic sophistication based on the current opponent and the strategic environment and 2) the existence of a form of *bounded rationality* in strategic thinking, which underlines the crucial role of cognitive operations and abilities for the implementation of sophisticated interactive decisions (Bilancini et al., 2019; Crawford, 2003; Goodie et al., 2012; Grosskopf & Nagel, 2008; Mazzocco et al., 2013). Experimental evidence supports the co-existence of both factors. Players with sufficient cognitive skills can adapt their level of strategic sophistication based on the predicted level of the opponent (Agranov et al., 2012; Alaoui et al., 2020; Alaoui & Penta, 2022). However, a notable proportion of players do not manage to increase sophistication when playing with highly sophisticated individuals, suggesting the presence of bounded rationality (Jin, 2021). Moreover, extensive behavioral evidence converged in showing that higher cognitive abilities (e.g., fluid intelligence, cognitive reflection, working memory) predict higher strategic sophistication in different types of games (Brañas-Garza et al., 2012; Burnham et al., 2009; Carpenter et al., 2013; Fehr & Huck, 2016; Hanaki et al., 2016; Jin, 2021; Kiss et al., 2016; Proto et al., 2019; Reverberi et al., 2022; Zonca et al., 2020b).

In recent years, experimental research investigating the possibility of *enhancing* individuals’ strategic sophistication has attracted growing interest. Improvements in players’ strategic sophistication have been found through feedback exposure (Gill & Prowse, 2016; Knoepfle et al., 2009; Marchiori et al., 2021; Selten et al., 2003), observational learning (Zonca et al., 2021), experience with alternative decision rules (Zonca et al., 2019) and step-by-step training (Verbrugge et al., 2018). In a recent eye-tracking study, Marchiori et al. (2021) showed that learning by experience in games depends on the learning environment, that is, the type of game structures provided in the learning stage. The authors revealed remarkable heterogeneity in learning rates, even if players received feedback in games requiring relatively simple cognitive operations (i.e., learning to form first-order beliefs). Interestingly, Gill and Prowse (2016) showed that higher cognitive abilities are associated with higher learning rates in repeated strategic interactions. Altogether, the few existing studies on learning in strategic interactions suggest that enhancing strategic sophistication is possible and that successful learning may be predicted by the interplay between exogenous (e.g., the learning environment) and endogenous (e.g., cognitive skills) factors. This hypothesis is consistent with theories of strategic sophistication (Alaoui & Penta, 2016; Frydman & Nunnari, 2021) supporting 1) a plastic notion of sophistication, whose level may be varied based on knowledge of the characteristics of the strategic context, and 2) a critical role of cognitive abilities, which may prevent players from increasing significantly their level of strategic thinking.

Nonetheless, our understanding of the factors and the contextual conditions shaping learning in strategic interaction is minimal, and further research is needed to unveil them. In particular, no study has clarified how the interaction between the type of learning environment and individual cognitive abilities can shape relevant learning processes in strategic interaction. In this study, we will focus on learning by feedback since it represents the most natural and common learning opportunity we encounter in real-life strategic interactions.

This study employs an experimental paradigm where participants engage in a series of 3x3 games presented in matrix form. The games are categorized into three classes of increasing complexity, requiring one, two, or three steps of recursive thinking to determine the optimal strategy. We consider a strategy optimal when the players reach the “Nash equilibrium”,^1^ the solution prescribed in classical game theory when the opponent is a rational utility maximiser. The experimental design involved two treatments: Feedback and Baseline. In the former, we investigated the emergence of learning by feedback in strategic interaction, whereas in the latter, feedback was not provided, but repeated exposure to the same classes of games of the Feedback treatment was maintained. Volunteers in both treatments faced three subsequent phases in which they played games with an artificial counterpart (henceforth, the *computer*) that applied a fixed (but unknown) strategy (i.e., the Nash equilibrium). In the Assessment phase, they played games belonging to the three different classes, without feedback. In the Learning phase, depending on the assignment to one of three experimental conditions, participants played a new series of games, all belonging to one game class from those faced in the Assessment phase. In this phase, participants in the Feedback treatment received feedback on the choice of the computer; in contrast, participants in the Baseline treatment did not receive any feedback. In the Reassessment phase, all participants played a new series of games of the three different classes, as in the first phase. By comparing participants’ behavior in the Assessment and Reassessment phases, we aim to assess whether feedback-based learning enhances their strategic sophistication. The use of different classes of games allows us to examine the emergence of different types of learning: in particular, we can test whether acquired knowledge is transferred only to strategic settings with identical structure (context-specific learning) or if it can be transferred to different ones (transfer of learning).

During all the experimental phases, we use mouse-tracking. Mouse-tracking is a process tracing technique revealing how individuals acquire and use available information in their decisions. Process tracing techniques, including mouse-tracking and eye-tracking, have been used successfully in previous research to disclose players’ resolution procedures and strategies in games, unveiling crucial sources of suboptimality and heterogeneity in strategic behavior (Coricelli et al., 2020; Konovalov & Ruff, 2022). More specifically, findings coming from a large body of research using mouse-tracking (Brocas et al., 2014; Camerer et al., 1993; Costa-Gomes & Crawford, 2006; Costa-Gomes et al., 2001; Evans & Krueger, 2011, 2014; Johnson et al., 2002) and eye-tracking (Arieli et al., 2011; Devetag et al., 2016; Meijering et al., 2012; Polonio & Coricelli, 2019; Polonio et al., 2015; Sickmann & Le, 2016; Stewart et al., 2016; Zonca et al., 2019, 2020a) have shown remarkable variability in patterns of information acquisition in interactive games, which in turn reveals the use of specific resolution strategies and explains heterogeneity and suboptimality in strategic choices. In the present study, participants directly selected by mouse-clicking the information of the game matrix (i.e., the payoffs) they wanted to consider in making their decision. We extracted relevant sequences of mouse clicks (Information Acquisition Sequences, IASs), which assess whether players performed specific procedures to solve particular classes of games. The analysis of IASs can disclose whether players change their resolution procedures and strategies after receiving feedback in a given environment.

To explore whether differences in cognitive abilities are associated with different learning patterns, we collected two individual measures: the Cognitive Reflection Test (CRT, Frederick, 2005; Primi et al., 2016) and the N-back task. The CRT has been introduced to measure the individual propensity to rely upon reflective or intuitive thinking styles (i.e., the so-called individual *cognitive reflection* level). It correlates with measures of intelligence (Otero et al., 2022), numeracy (Campitelli & Gerrans, 2014; Del Missier et al., 2012; Otero et al., 2022), decision-making (Burks et al., 2009; Christelis et al., 2010; Graffeo et al., 2015), reasoning (Hoppe & Kusterer, 2011; Oechssler et al., 2009), learning (Don et al., 2016), and response inhibition (Campitelli & Gerrans, 2014; Del Missier et al., 2012). Previous evidence showed that higher CRT scores predicted higher levels of strategic sophistication in different types of games (Brañas-Garza et al., 2012; Carpenter et al., 2013; Fehr & Huck, 2016; Kiss et al., 2016; Zonca et al., 2020b). Another process that might play a role in determining the success of learning is linked to the ability to maintain and manipulate pieces of information in working memory, which could be necessary to apply recursive reasoning and multiple steps of strategic thinking (Allred et al., 2016; Camerer et al., 2004; Carpenter et al., 2013; Rydval et al., 2009; Sher et al., 2014). The N-back task represents a specific measure to target such processes of retention and manipulation of information in working memory (Conway et al., 2005; Kane & Engle, 2002). For the current experiment, both 2- and 3-back tasks were used.

The contribution of this experimental work is threefold. First, we provide new insights into the learning process in strategic interaction, focusing on *how* the learning environment can shape different types of learning mechanisms, i.e., context-specific learning and transfer of learning. Second, we test whether such learning processes are modulated by individual differences in cognitive abilities. Third, leveraging the mouse-tracking technique, we can disclose the cognitive operations modified or acquired through learning.

## HYPOTHESES

Our hypotheses rest on theoretical and empirical works (Alaoui & Penta, 2016; Frydman & Nunnari, 2021; Jin, 2021; Marchiori et al., 2021) supporting 1) a notion of *plasticity* of strategic sophistication that depends on the characteristics of the strategic environment and 2) a critical role of cognitive bounds (i.e., bounded rationality) in the emergence of high levels of strategic thinking.

We propose two general hypotheses. First, feedback exposure can enhance understanding of the strategic environment and opponent behavior, prompting exploration of more effective resolution strategies. However, learning in a single environment may restrict the exploration of alternative strategies and higher-order reasoning procedures if these are not required in that specific context, potentially hindering successful adaptation to diverse interactive scenarios. Second, higher cognitive abilities enable or enhance these learning processes. We hypothesize that learning from feedback is rooted in the ability to extract and comprehend relevant behavioral patterns. Thus, we expect individual levels of cognitive reflection to predict learning outcomes in our games. Additionally, learning to play strategically (i.e., applying recursive reasoning and multiple steps of strategic thinking) may demand a high working memory capacity. Consequently, we hypothesize that working memory abilities modulate feedback-based learning.

Specifically, our experimental study aims to test the following hypotheses:

1. **Players can increase their strategic sophistication through feedback.**
  Exposure to feedback (and not mere repeated exposure to games) increases strategic sophistication.
2. **The learning context modulates the enhancement of strategic sophistication.**
  2a.  **Context-specific learning.** Exposure to feedback in specific learning contexts increases sophistication in *similar* strategic settings.
  2b.  **Transfer of learning.** The knowledge acquired through feedback can be transferred to other *different* strategic contexts.
3. **Sophistication and learning in games is modulated by individual cognitive abilities.**
  3a.  **Higher cognitive abilities support strategic sophistication.** Higher levels of cognitive reflection and working memory predict a higher proportion of Nash equilibrium choices, especially in game classes requiring mentalizing and multiple steps of strategic thinking.
  3b.  **Higher cognitive abilities support context-specific learning.** Higher levels of cognitive reflection and working memory predict higher rates of context-specific enhancement of strategic sophistication.
  3c.  **Higher cognitive abilities support the transfer of learning.** Higher levels of cognitive reflection and working memory predict the transfer of acquired knowledge to novel strategic contexts.
  3d.  **Higher cognitive abilities predict learning in complex strategic settings.** The impact of cognitive abilities (cognitive reflection, working memory) on learning is stronger in complex strategic settings (i.e., entailing the formation of second-order beliefs or multiple steps of recursive thinking).
4. **Players learn *resolution strategies* linked to the experienced learning environment.**
  4a.  **Learning by feedback changes players’ resolution strategies.** Exposure to feedback in a learning environment leads to the exploration and acquisition of new patterns of information encoding and representation, which are re-applied in similar and different strategic settings.
  4b.  **Higher cognitive abilities support learning of resolution strategies.** Changes in patterns of information encoding and representation are predicted by higher levels of cognitive reflection and working memory.

## MATERIALS AND METHODS

### The Games

We used 75 two-person 3 × 3 one-shot matrix games^2^ with a unique Nash equilibrium, divided into three classes of games differing in their structure and level of complexity, namely 1-step, 2-step, and 3-step (see Figure 1 for examples of the three classes of games). Within each game class, we varied the payoffs’ magnitude and location, keeping the game structural properties described below constant:

-  **1-step games**: The participant has a “strictly dominant” strategy. A strictly dominant strategy ensures a higher payoff than every other strategy (i.e., the “dominated” strategies), regardless of what the opponent does. Consequently, the optimal solution can be detected through one step of strategic thinking (i.e., eliminating the dominated strategies) by simply reasoning on own payoffs, even without considering the counterpart’s incentives and forming beliefs about the opponent’s actions.
-  **2-step games**: The *opponent* has a “strictly dominant” strategy. Therefore, the player needs to identify the opponent’s dominant strategy and then best respond to its expected action (first-order belief). Two steps of strategic thinking (or two steps of iterated elimination of dominated strategies) are thus required to solve this type of game.
-  **3-step games**: The solution can be found with three steps of strategic thinking. First, participants should realize that they have a “dominated” strategy, meaning that such a strategy is always worse off than another participant’s strategy, independently of the potential action of the opponent. Participants should believe that the opponent would know that the participant will not choose the dominated strategy. Therefore, the counterpart would choose its best action by only considering the two remaining rows. After excluding one row, the opponent has a dominant action. The participant should identify this action and best respond to it. This is a typical example of a game requiring three steps of reasoning (or 3 steps of iterative elimination of dominant/dominated strategies) where the participant has to form second-order beliefs.

In sum, increasingly higher strategic sophistication is required to solve 1-step, 2-step, and 3-step games. 1-step games do not require the formulation of beliefs about the opponent’s moves or expectations about our potential actions, and the solution of the game can be reached just by reflecting on one’s own incentives. 2-step games require the formulation of first-order beliefs, that is, forming beliefs about the opponent’s move by analyzing the counterpart’s incentives and best responding to them. 3-step games require the formulation of second-order beliefs, i.e., forming beliefs about the opponent’s expectations regarding our actions.

### Participants and Experimental Procedure

244 participants participated in the experiment (118 males, 126 females, mean age: 23.5, *SD*: ±4.22, range: 18–56). Volunteers were undergraduate or graduate students of the University of Milano-Bicocca who took, on average, 67.1 minutes (*SD*: ±16.33, range: 31.78–133.38) to complete the experiment. The local Ethics Committee approved the study.

**Figure 2.**
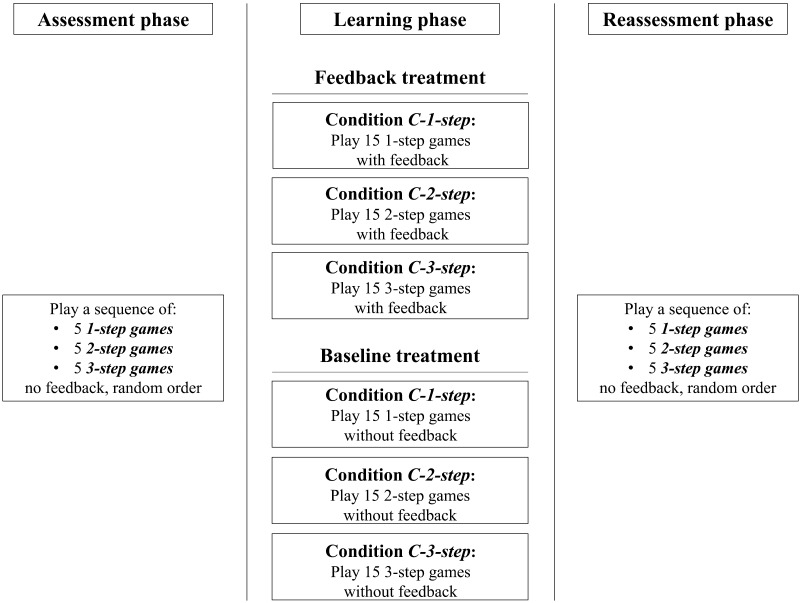
Schematic representation of the experimental design. Participants faced 45 matrix games in three subsequent phases (Assessment, Learning, and Reassessment). Depending on the assigned experimental condition, volunteers played one type of game in the Learning phase (C-1-step, C-2-step, or C-3-step conditions) with (Feedback treatment) or without (Baseline treatment) feedback. In the Assessment and Reassessment phases, five 1-step, five 2-step, and five 3-step games were presented in fully random order (independently of the game class) and feedback was never provided. Stimuli were selected to ensure participants played each game only once.

First, participants faced a series of 45 games presented in matrix form, in which they played with a *computer* that always played the Nash equilibrium strategy. Participants were told that the computer would aim to maximize its own earnings and would keep the same strategy throughout the experiment, independently of the participants’ choices.^3^ Participants faced three phases of 15 games each: Assessment, Learning, and Reassessment (Figure 2). Experimental subjects were randomly assigned to one of two between-subject treatments and one of three between-subject conditions. In the Feedback treatment, participants were provided with feedback on the computer’s choice in the Learning phase, whereas in the Baseline treatment feedback was never provided. The *condition* defined the type of game (i.e., the strategic context) faced in the Learning phase. In Condition-1-step (C-1-step), Condition-2-step (C-2-step), and Condition-3-step (C-3-step) conditions, participants played 1-step, 2-step or 3-step games, respectively, in the Learning phase. In both the Assessment and Reassessment phases, participants played five games of each game class presented in random order independently of the game class and without feedback (Figure 2). Participants were *not* informed about the existence of different classes of games in the three experimental phases.

Concerning the Feedback treatment (*N* = 169), 57 participants were assigned to the C-1-step condition, 56 to the C-2-step condition, and 56 to the C-3-step condition. In the Baseline treatment (*N* = 75), which served as a control treatment, 25 participants were assigned to the C-1-step condition, 25 to the C-2-step condition, and 25 to the C-3-step condition.

In total, 13 experimental sessions were carried out. Experimental sessions included a minimum of 9 and a maximum of 23 volunteers. Participants arrived at the lab at a given time and were provided by the experimenter with a number, referring to a computer workspace. Positions were assigned to ensure that at least one place between a workstation and another was left free. Moreover, participants were asked not to talk to each other and not to make noise during the experimental procedure. The computers were connected to a host server administered by the experimenter. Stimuli were displayed with a custom-made program implemented with Psychopy (Peirce et al., 2019) through a computer screen with a resolution of 1920 × 1080 and a dimension of 27 inches. Once sat in front of the assigned workspace, participants were asked to carefully read the instructions and ask questions if they had any doubts or uncertainties. Control questions were administered to ensure that all the information was fully understood. Before starting to play the games, volunteers had to wait until all the participants completed the comprehension test, as checked by the experimenter. To allow participants to familiarize themselves with the stimuli and the experimental procedure, two training games were presented at the beginning of the Assessment phase, and an additional training game was provided at the start of the Learning phase. Subjects were always kept aware of the phase they were completing, and, at the end of each phase, they were reminded of the upcoming phase.

At the end of the experimental procedure, three games (one for each phase) were randomly selected, and participants were paid according to their choices matched with the actual computer’s decisions. Ten points of the matrix payoff corresponded to a payment of 0.70 euros. The total reward consisted of the three extracted game rewards plus the earnings from the *n*-back tasks. Participants earned between 6 and 17.5 euros (mean: 12.57, *SD*: ±1.94).

### Experimental Task and Mouse-Tracking Procedure

**Figure 3.**
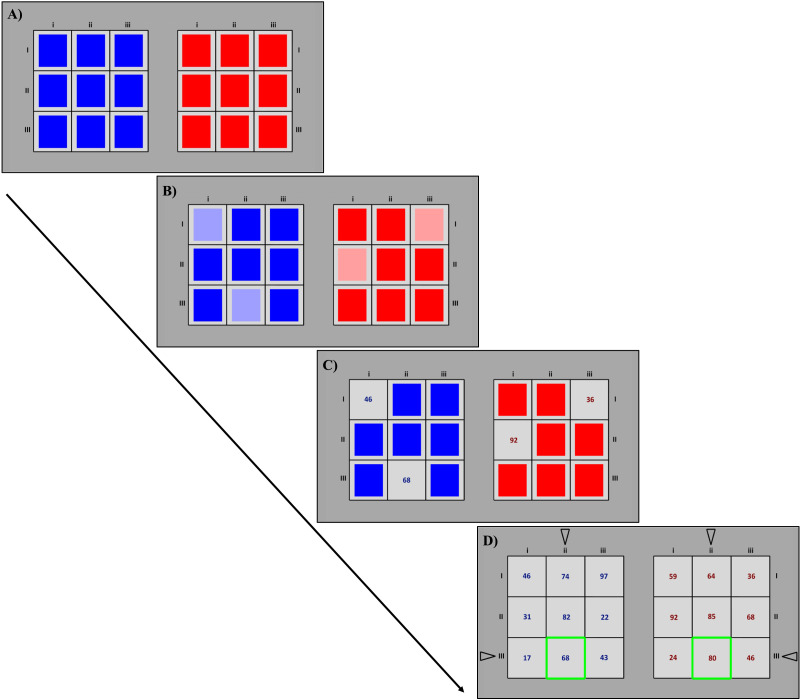
Example of a typical trial, i.e., a game. **A.** Initially, the matrices’ content is covered by colored boxes (blue for the Own-payoffs matrix, red for the Other-payoffs matrix). **B.** Participants select the cells of interest with the mouse to explore their payoffs (maximum 6 at a time). **C.** Payoffs in the selected cells are revealed. When the participant is ready to make her choice, a row is selected by key press. **D.** In the Learning phase (and Feedback treatment) only, participants receive feedback on the choice of the computer and the outcome of the game (i.e., the combination of their own and the computer’s choice). The arrows indicate each player’s choice, whereas the green frame highlights the outcome by combining the participant and the computer choices (row III and column ii in the present example).

In each of the total 45 trials, participants played a single interactive game with the computer.^4^ The games were displayed through two matrices next to each other (Figure 3A), one containing the participant’s payoffs (Own-payoffs matrix) and the other the computer’s payoffs (Other-payoffs matrix). Hence, we could disentangle participants’ exploration of their own and the computer’s payoffs using mouse-tracking. The cells and the two matrices, showing the participants’ and the computer’s payoffs, were displayed at an optimal distance from each other to ensure disentangling subjects’ goal-directed actions (i.e., mouse clicks). All participants played the role of the “row player,” choosing one of the three rows in the Own-payoffs matrix by pressing a key button. At the same time, participants knew that the computer would take the role of the “column player,” choosing one of the three columns of the Other-payoff matrix. Cells’ content was covered by colored boxes, either blue (the participant’s payoffs) or red (the computer’s payoffs). To explore the payoffs, participants used the mouse. The cells of interest had to be selected by pressing the left button (Figure 3B) and then opened through a right button (Figure 3C) press. Participants could open up to six cells simultaneously and could observe up to six cells (i.e., payoffs) at a time. To open further cells in another iteration, participants first need to close the cells opened in the previous iteration. Allowing the opening of multiple cells simultaneouslty decreased the potential operational cost associated with single-cell opening. The limit in the number of cells that could be open at the same time (6 cells) guaranteed the possibility of extracting cognitively meaningful units of process data (IASs, see the Process Data Analysis section). For instance, opening 6 cells allows participants to compare two actions (e.g., two rows or two columns) to identify the best choice among the two or detect the presence of a dominant/dominated action. On the other hand, the 6-cells limit prevents the acquisition of all information in one single iteration, by opening all the 18 cells available. Before deciding, participants could repeat the exploration procedure as often as they wanted, exploring all the matrix information. No time limit was set. The computer’s payoffs were displayed in red, whereas participants’ payoffs were in blue. Row and column numbers were always presented next to the matrices. When participants made their choices, an arrow appeared pointing to the selected row. All the matrix content was then displayed and remained available until subjects decided to move to the subsequent trial by mouse click. In the Feedback treatment, feedback in the Learning phase consisted of a black arrow in correspondence to the computer’s strategy and a green frame highlighting the cell resulting from the combination of the two players’ choices (Figure 3D). Feedback was displayed only once participants made their decisions.

### N-Back Task

Working memory abilities were assessed through the N-back task. Participants were presented with a sequence of single letters appearing in the middle of the screen for 1000 ms. In two different sessions, they were asked to evaluate, by pressing a key bottom, whether the letter in the current trial matches the letter presented two (2-back task) or three (3-back task) trials before. For the present experiment, both capital and lowercase letters were employed. Between the occurrence of two letters, a fixation cross was displayed for 1000 ms. After practicing with a block of 16 trials provided with feedback, volunteers faced the 2- and 3-back tasks. They were informed that they would have been rewarded 0,02 euros for each correct trial, for a maximum achievable payoff of 2,00 euros for both tasks (2- and 3-back tasks consisted of 100 trials each).

### CRT

Participants, after completing the N-back tasks, underwent the CRT. We employed the 6-item CRT-long version (CRT-L) (Primi et al., 2016) to overcome the limitations of the original tool (Frederick, 2005), consisting of only 3 items. The CRT-L has been proven to better discriminate between individuals with different levels of cognitive reflection ability (Primi et al., 2016).

In our experiment, each of the six CRT questions was displayed individually on the screen (see the Supplementary Information for the full items list). Volunteers had to type their responses in a box. No time limit was imposed, and participants were not rewarded for their performance to avoid interfering through incentivization with the participants’ individual thinking dispositions.

### Behavioral Data Analysis

First, we addressed hypotheses 1 and 2 (2a, 2b) by testing for the presence of differences in the implementation of Nash equilibrium choices across four main factors of interest and their interactions: treatment (Feedback; Baseline), condition (C-1-step; C-2-step; C-3-step), game class (1-step; 2-step; 3-step), and phase (Assessment; Reassessment). **Hypothesis 3** (3a, 3b, 3c, 3d) has been tested considering two additional factors: the individual cognitive reflection level (CRT) and the individual working memory level (N-back). To facilitate the interpretation and visualization of results, we divided our participants into two groups across each cognitive measure to assess and analyze the interaction between specific cognitive abilities and learning outcomes. Concerning the CRT, we divided participants with high cognitive reflection (High-CRT: CRT score > 3) from participants exhibiting low cognitive reflection (Low-CRT: CRT score ≤ 3) by median split. To test the influence of working memory abilities on learning, we computed the mean scores for the 2- and 3-back tasks, and participants from both treatments were split according to the median value into a High-WM and Low-WM group (High-WM: N-back score > 67; Low-WM: N-back score ≤ 67). Nevertheless, we replicated the main analyses concerning cognitive abilities using the CRT score and the N-back score as continuous variables.

The statistical approach entailed mixed-effects logistic models with trial-by-trial Nash choice (0: choice inconsistent with Nash equilibrium; 1: choice consistent with Nash equilibrium) as binary dependent variable. In all models, the intercept was allowed to vary across participants, including a random effect on the intercept at the participant level to account for intra-subject correlations of repeated assessments.

### Process Data Analysis

**Figure 4.**
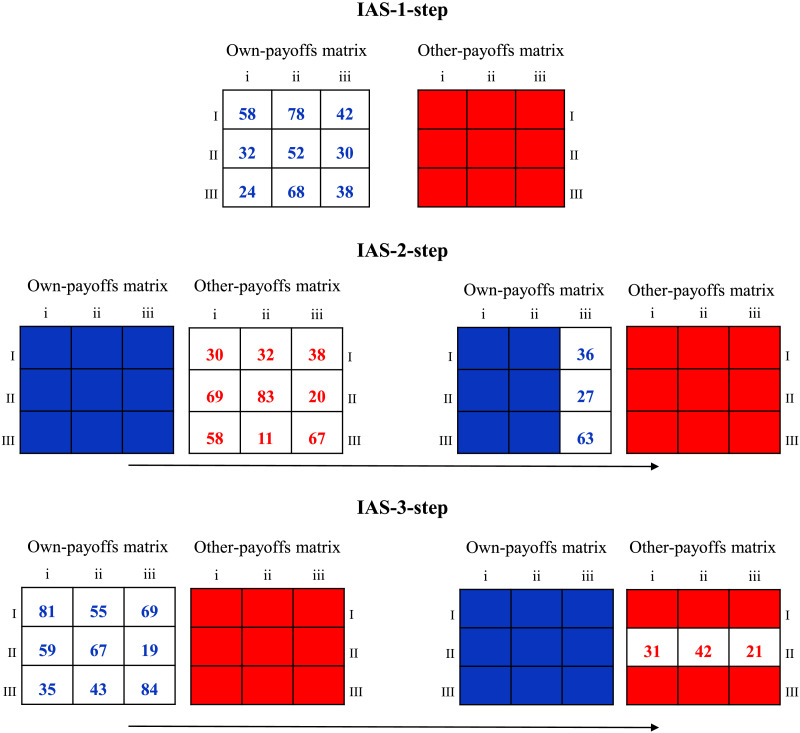
Graphical representation of IAS-1-step, IAS-2-step, and IAS-3-step. Cells not covered by colored boxes indicate the cells that should be opened to achieve each IAS. Opening of specific cells must follow a specific order, as described next. **IAS-1-step**: To complete the IAS-1-step, participants have to open all the cells of the Own-payoffs matrix without opening more than 3 cells of the Other-payoff matrix. **IAS-2-step**: First, the IAS-2-step requires opening all the cells of the Other-payoffs matrix without opening more than 3 cells of the Own-matrix payoffs. Then, participants need to open at least one column of the Own-payoffs matrix (in the present example, selected cells in the matrix displayed on the right highlight one of the three possible columns that can be opened to complete the IAS). **IAS-3-step**: To complete this IAS, participants first need to open all the cells of the Own-payoffs matrix, without opening 3 or more cells of the Other-payoffs matrix. Then, participants should attend to at least one row of the Other-payoffs matrix (opened cells in the matrix displayed on the right represent one of the three possible rows that can be opened to complete the IAS).

To investigate the relationship between lookup patterns, strategic behavior, and learning, we considered three main information acquisition sequences (IASs) reflecting the ordered cognitive and strategic procedures that are critical in the resolution of our three classes of games (Figure 4).^5^ We highlight that IASs do not aim to express exhaustive ordered sequences of mouse clicks that guarantee the reaching of the Nash equilibrium solution in different classes of games. Instead, IASs are ordered information acquisition steps underlying critical cognitive operations that should emerge during game resolution when increasingly sophisticated strategic thinking is applied. Indeed, 1-step games require a complete exploration of players’ own incentives (IAS-1-step); 2-step games require the formation of first-order beliefs, a critical process that is represented by the IAS-2-step; 3-step games require the formation of second-order beliefs, which is expressed by the IAS-3-step. In each game, we considered whether the participant had completed the three IASs, therefore disclosing which strategic thinking operations were performed by the participant in that game. IASs were computed as follows:

-  **IAS-1-step**: This IAS reflects a complete information acquisition of own payoffs, which is helpful for detecting dominant and dominated actions for the participant. By allowing the identification of the participant’s dominant action, the achievement of this IAS supports the resolution of 1-step games.
  To complete this IAS, participants must explore all nine payoffs of the Own-payoffs matrix. However, to complete this IAS, participants can explore a maximum of 3 cells of the Other-payoff matrix before or during the opening of the nine payoffs of the Own-payoff matrix. This constraint has been introduced to prevent the attainment of IAS-1-step through the implementation of a common heuristic (i.e., coordination) involving the comparison between the payoff located in the two matrices, which is incompatible with the identification of the participant’s dominant action (see Polonio et al., 2015; Devetag et al., 2016; Zonca et al., 2019).
-  **IAS-2-step**: To complete this IAS, participants must initially open all the cells of the Other-payoffs matrix; before or during the opening of the nine payoffs of the Other-payoffs matrix, participants can explore a maximum of 3 cells of the Own-payoffs matrix. Then, participants need to open at least one column of the Own-payoffs matrix. This IAS reflects the formation of first-order beliefs about the potential action of the opponent and the subsequent identification of the best response to such prediction, which are the two necessary steps to play Nash equilibrium in 2-step games.
-  **IAS-3-step**: To complete the IAS-3-step, participants must first attend all the payoffs in the Own-payoffs matrix (i.e., completing the IAS-1-step). Then, participants need to attend at least one row of the Other-payoffs matrix to identify the computer’s best response to the participant’s predicted action. This IAS reflects the implementation of a thinking process involving second-order beliefs, which are fundamental to solving 3-step games. Players forming second-order beliefs should first analyze their own payoffs to predict the beliefs of the counterpart (about the participant’s choice), and then move to the computer’s payoffs to predict the computer’s best response to its beliefs. The IAS-3-step is not strictly sufficient to find the equilibrium solution in 3-step games. In fact, since 3-step games contain a dominated action for the row player, an optimal pattern of information acquisition entails the opening of the two undominated rows of the Other-payoff matrix to spot the subsequent computer’s dominance. However, the IAS-3-step is meant to represent the process of formation of second-order beliefs (critical for the resolution of 3-step games), rather than the complete path of resolution of 3-step games. Specifically, in the context of 3-step games, we did not require identifying and excluding the row player’s dominated action as constitutive of second-order reasoning.

For each trial, we registered for each of the three IAS whether it has been applied (1) or not (0). These three trial-by-trial binary variables were used as dependent variables, in three different mixed-effect logistic models (random effect on the intercept at the subject level) to test for changes in information acquisition patterns across phases, conditions, game classes, cognitive levels, and their interactions. Concerning cognitive abilities, we restricted the analysis of IASs to the cognitive measures modulating learning effects in the behavioral analyses.

### Data Exclusion

Before running the analyses, we computed the average of opened cells in each experimental phase for every participant. We excluded those subjects (*N* = 14)^6^ who did not reach, in each phase, an average of opened cells greater or equal to six. Such threshold corresponds to the number of cells a participant could open simultaneously with a single mouse click. We considered six cells (e.g., two rows or two columns) as the minimum amount of information a player should acquire to make a meaningful decision (e.g., choosing the best between two available rows or columns). Therefore, we aimed to exclude those participants who did not attend, on average, to this minimum amount of evidence to make their decisions, *in each phase*. This criterion was also meant to control for potential between-phase differences arising from marked discrepancies in the effort devoted in different phases of the task (e.g., due to fatigue). Therefore, after data exclusion, data analysis was conducted with a total sample of *N* = 230 experimental subjects, divided into Feedback (*N* = 159) and Baseline (*N* = 71) treatments.

However, we acknowledge that the main results did not differ when including the entire sample (see Supplementary Information, Supplementary Results, for the main descriptive statistics of the entire sample).

## RESULTS

### Learning by Feedback and the Impact of the Learning Environment

In this section, we test hypotheses 1 and 2 by investigating the emergence of learning by feedback in strategic interaction and the role of the learning context in such learning effects.

To investigate the occurrence of learning effects, we employed a mixed-effects logistic model (Model 1) on choice data. This model examined how learning effects depend on feedback presence in the Learning phase, the learning context, and the strategic environment participants encountered. We used trial-by-trial Nash equilibrium choices as the binary dependent variable (0: inconsistent with Nash equilibrium; 1: consistent with Nash equilibrium). Independent factors included treatment (Feedback; Baseline), condition (C-1-Step; C-2-step; C-3-step), game class (1-step; 2-step; 3-step), phase (Assessment; Reassessment), and their interactions. We included participants as a random effect on the intercept. In the main text, we focus on the main effect of phase and its interactions with other predictors to highlight the factors modulating learning in strategic behavior. Nonetheless, complete results can be found in the Supplementary Information (Table S2, S3).

Omnibus results (Table S2) show a significant main effect of phase (*χ*2(1, *N* = 230) = 5.82, *p* = .016) and a significant interaction between phase and treatment (*χ*2(1, *N* = 230) = 8.64, *p* = .003). The interaction is driven by a significant effect of phase in the Feedback treatment (*B* = 0.35, *z* = 5.10, *p* < .001), where the proportion of Nash choices increases from 52% in the Assessment to 59% in the Reassessment phase, across all conditions and game classes. On the contrary, we do not observe a significant effect of phase in the Baseline treatment (*B* = −0.03, *z* = −0.31, *p* = .757), where the proportion of Nash choices slightly decreases from 53% to 52% in the Reassessment phase. These first results support **Hypothesis 1**, revealing the presence of learning by feedback.

**Figure 5.**
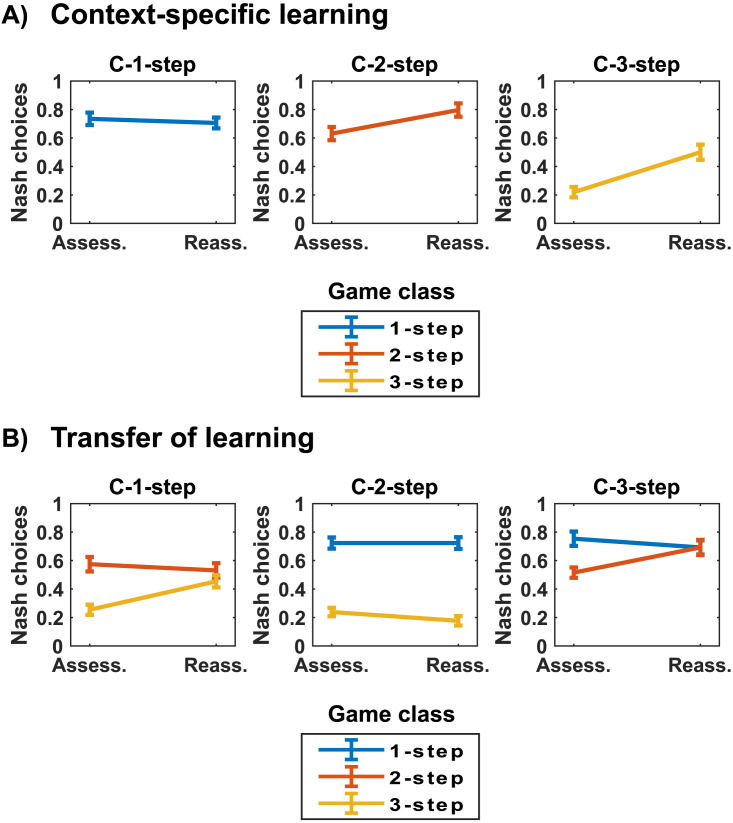
**Effects of context-specific learning and transfer of learning (Feedback treatment). (A) Context-specific learning**. We plot the proportion of Nash choices across phases (Assess.: Assessment; Reass.: Reassessment) in each of the three conditions (C-1-step, C-2-step, C-3-step) of the Feedback treatment. Results refer to choices made in the same game class faced in the Learning phase (C-1-step: 1-step games; C-2-step: 2-step games; C-3-step: 3-step games). **(B) Transfer of learning**. Here, we consider choices in game classes that players have not faced in the Learning phases in order to test the presence of transfer learning effects.

We also observe a significant three-way interaction between treatment, condition, and phase (*χ*2(2, *N* = 230) = 10.15, *p* = .006) and a significant four-way interaction between treatment, condition, game class, and phase (*χ*2(4, *N* = 230) = 13.05, *p* = .011), suggesting that learning *by feedback* in specific learning environments determines whether and how acquired knowledge can be transferred to new strategic environments. In particular, results show the emergence of significant effects of context-specific learning (**Hypothesis 2a**) and transfer of learning (**Hypothesis 2b**). These effects are displayed in Figure 5. Relevant contrasts are reported in Table S3.

First, players in the Feedback treatment increase their sophistication in those games encountered in the Learning phase (*context-specific learning*, Figure 5A). Indeed, players in the Feedback treatment increase their proportion of Nash choices in 2-step games in the C-2-step condition (from 63% to 80%, Figure 5A, middle panel) and in 3-step games in the C-3-step condition (from 22% to 50%, Figure 5A, right panel). We do not observe learning effects in 1-step games in the C-1-step condition (Figure 5A, left panel), in which sophistication is already high in the Assessment phase (74%). Learning effects observed in the Feedback treatment are absent in the Baseline treatment (Figure S2A, S2B, S2C, right panels, Supplementary Information). These results support **Hypothesis 2a**, suggesting that exposure to feedback in a learning context enhances sophistication in future interactions in similar strategic settings.

Second, results reveal the emergence of *transfer of learning* by feedback (Figure 5B). Exposure to feedback in 1-step games (C-1-step condition) increased sophistication (from 26% to 46%) in 3-step games (Figure 5B, left panel). In this regard, we highlight that 1-step and 3-step games share the first step, i.e., the need to detect dominance properties (i.e., dominant or dominated actions) in the Own-payoffs matrix. The observed transfer-learning effect suggests that receiving feedback in 1-step games triggered the application of relevant procedures for detecting *dominated* actions in 3-step games, which in turn led to the formation of second-order beliefs (“the computer expects me not to choose the dominated action”) and, consequently, to a significant improvement in 3-step games. Moreover, receiving feedback in 3-step games (C-3-step condition) favored learning in 2-step games (from 52% to 69%. Figure 5B, right panel). This result suggests that exposure to feedback in complex games requiring multiple steps enhances sophistication in relatively simpler games. Importantly, no transfer learning effects are observed in the Baseline treatment (Figure S2A, S2B, S2C, right panels, Supplementary Information). Altogether, these transfer learning effects suggest that relevant knowledge acquired through feedback can be transferred to novel strategic environments, in line with **Hypothesis 2b**.

### The Role of Cognitive Abilities in Strategic Sophistication and Learning by Feedback

#### Cognitive Abilities and Strategic Sophistication.

We tested **Hypothesis 3a** by investigating whether heterogeneity in strategic sophistication in the Assessment phase (before any learning opportunities) could be explained by inter-individual variability in cognitive abilities (cognitive reflection and working memory).^7^

**Figure 6.**
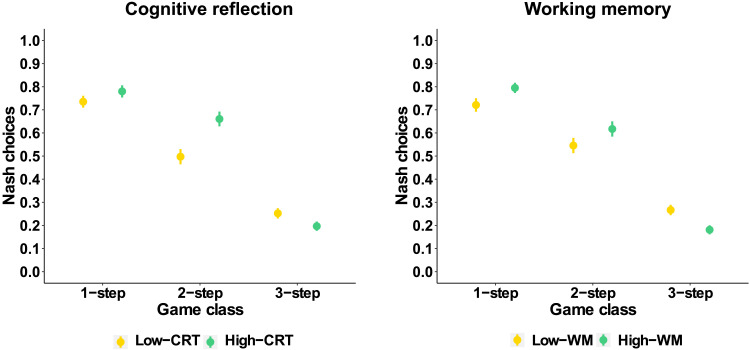
Proportion of Nash choices in the Assessment phase, plotted by level of cognitive ability (left: Cognitive reflection, Low-CRT vs. High-CRT; right: Working memory (N-back score), Low-WM vs. High-WM) and along different classes of games (1-step, 2-step, 3-step). Error bars represent between-subject standard errors.

First, we ran a mixed-effect logistic model (Model 2) with trial-by-trial Nash equilibrium choices in the Assessment phase as the dependent variable, and cognitive reflection level (High-CRT: *N* = 119; Low-CRT: *N* = 111), game class and their interactions as independent factors. We consider data from Feedback and Baseline treatments since the Assessment phase is not influenced by the differences between treatments characterizing the Learning phase. Results of Model 2 (Figure 6, left panel) reveal significant effects of game class (*χ*2(2, *N* = 230) = 599.23, *p* < .001) and interaction between game class and cognitive reflection level (*χ*2(2, *N* = 230) = 32.57, *p* < .001). The effect of the cognitive reflection level does not reach significance (*χ*2(1, *N* = 230) = 3.50, *p* = .061). Simple effects (Table S4) reveal that players with high cognitive reflection show a higher proportion of Nash choices in 2-step games (Low-CRT: 50%; High-CRT: 66%) but exhibit a lower rate of Nash choices than Low-CRT players in 3-step games (Low-CRT: 25%; High-CRT: 20%). The positive effect in 1-step games does not reach significance (Low-CRT: 74%; High-CRT: 78%).

Second, we ran another mixed-effect logistic model (Model 3) similar to Model 2 but with the WM-level (High-WM: *N* = 116; Low-WM: *N* = 114) and respective interactions in place of the CRT-level as predictors. Results of Model 3 (Figure 6, right panel) reveal significant effects of game class (*χ*2(2, *N* = 230) = 601.33, *p* < .001) and interaction between game class and WM-level (*χ*2(2, *N* = 230) = 26.68, *p* < .001). The main effect of the WM-level is not significant (*χ*2(1, *N* = 230) = 0.42, *p* = .519). Simple effects (Table S5) reveal that players with high working memory show a higher proportion of Nash choices in 1-step games (Low-WM: 72%; High-WM: 79%), 2-step games (Low-WM: 55%; High-WM: 62%), but exhibit a lower rate of Nash choices than Low-WM players in 3-step games (Low-WM: 27%; High-WM: 18%).

Our results indicate that higher cognitive abilities are associated with increased rates of Nash equilibrium choices in 1-step and 2-step games (**Hypothesis 3a**). This association does not extend to 3-step games, in which the proportion of Nash choices in 3-step games is extremely low across all cognitive levels.

#### Cognitive Abilities and Sophistication Enhancement.

Building on our initial findings, which indicated that learning opportunities were only present in the Feedback treatment, we focused our subsequent analysis on this group to explore the role of cognitive abilities in driving learning outcomes. Specifically, we investigated how individual cognitive abilities influence learning and interact with the learning context to modulate enhancements in strategic sophistication.

First, we analyzed the role of *cognitive reflection* in strategic sophistication enhancement. We ran a mixed-effects logistic model (Model 4) with trial-by-trial Nash equilibrium responses as the binary dependent variable and learning condition, game class, phase, cognitive reflection level, and their interactions as independent factors. We included participants as a random effect on the intercept. Omnibus results (Table S6, Supplementary Information) reveal a significant interaction between cognitive reflection level and phase, suggesting a role of cognitive reflection (*χ*2(1, *N* = 159) = 20.26, *p* < .001) in the emergence of learning by feedback. Simple effects reveal an increase in the proportion of Nash choices from the Assessment to the Reassessment phase in High-CRT participants (from 55% to 68%, *B* = 0.739, *z* = 6.54, *p* < 0.001), while learning is absent in Low-CRT participants (from 49% to 51%, *B* = 0.093, *z* = 1.05, *p* = 0.295).

**Figure 7.**
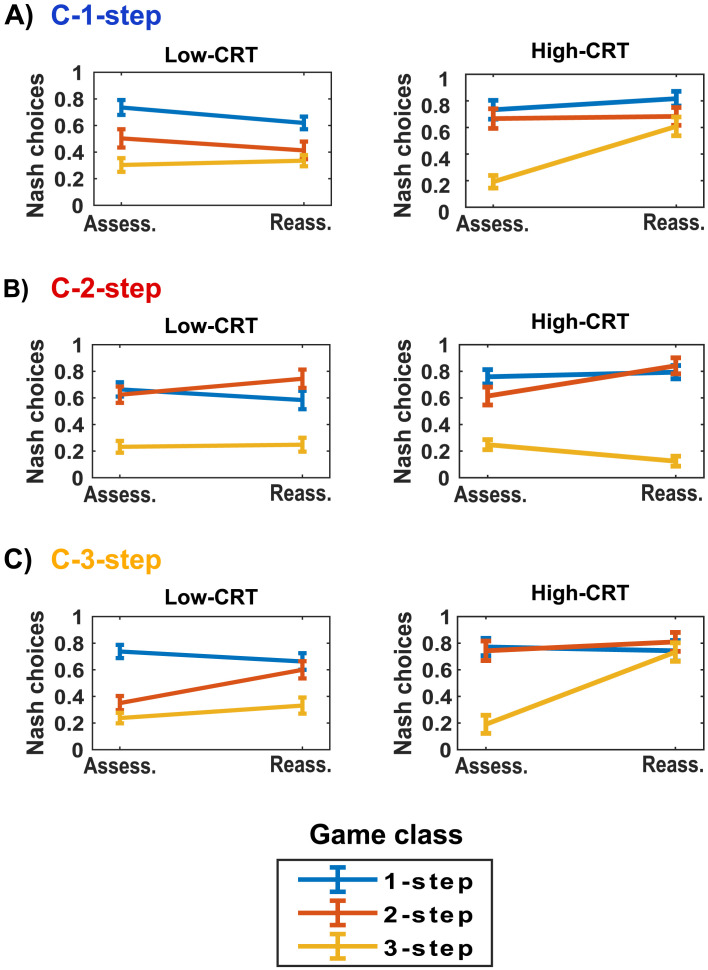
**The interplay between learning context, strategic environment, and cognitive reflection level in modulating learning by feedback (Feedback treatment)**. Proportion of Nash choices for High-CRT and Low-CRT participants, across phases (Assess.: Assessment; Reass.: Reassessment), game class (1-step, 2-step, 3-step games) in each of the three experimental conditions (A: C-1-step, B: C-2-step, C: C-3-step). Error bars represent between-subject standard errors of the mean. We use color coding to facilitate the readability of this figure and the identification of context-specific and transfer of learning effects across cognitive reflection levels. Indeed, each condition label (e.g., C-1-step) is associated with a color (e.g., blue) that corresponds to the color of the game class played in the Learning phase of that specific condition (e.g., blue). Therefore, combinations of conditions and game classes matching in color represent context-specific learning, whereas combination with unmatched colors expresses a transfer of learning effects.

Then, we tested whether the effect of cognitive reflection level interacted with the learning condition and the strategic environment. We observe a significant four-way interaction between cognitive reflection level, condition, game class, and phase (*χ*2(4, N = 159) = 35.64, *p* < .001). This interaction reveals that the players’ cognitive reflection level modulates the emergence of the context-specific and transfer of learning effects observed in Model 1. Results are displayed in Figure 7. Statistical analyses and regression tables of relevant contrasts are reported in the Supplementary Information (Table S7).

Concerning *context-specific* learning effects, in all the experimental conditions (Figure 7A, 7B, and 7C), High-CRT participants tend to increase their proportion of Nash choices more than Low-CRT participants when playing the same games encountered in the Learning phase. In the C-1-step condition, High-CRT participants increase their sophistication in 1-step games (from 73% to 82%, Figure 7A, right panel). In comparison, Low-CRT players decrease it (from 74% to 62%, Figure 7A, left panel), leading to a significant interaction (*B* = 1.104, *z* = 2.65, *p* = 0.008). In the C-2-step condition (Figure 7B), both High-CRT and Low-CRT participants increase their level of strategic thinking in 2-step games (High-CRT: from 63% to 84%; Low-CRT: 63% to 75%, interaction: *B* = 0.601, *z* = 1.43, *p* = 0.152). In the C-3-step condition (Figure 7C), High-CRT participants learn much more than Low-CRT ones (High-CRT: from 19% to 77%; Low-CRT: from 24% to 33%; interaction: *B* = 2.647, *z* = 5.68, *p* < 0.001). These results suggest that cognitive reflection supports context-specific learning, in line with **Hypothesis 3b**. Moreover, they suggest that high levels of cognitive reflection are required to learn to optimize behavior in complex strategic settings involving complex mentalizing procedures (i.e., second-order beliefs and 3-steps of strategic thinking, as required in 3-step games), in line with **Hypothesis 3d**.

Participants’ cognitive reflection level also impacts the *transfer of learning* effects (Table S7, Supplementary Information). Indeed, we observe a higher learning effect (*B* = 1.964, *z* = 4.82, *p* < 0.001) in High-CRT participants (compared to Low-CRT participants) in 3-step games when exposed to 1-step games in the Learning phase (C-1-step condition). More specifically, this learning effect is remarkable in High-CRT participants (19% to 61%, Figure 7A, right panel) and negligible in Low-CRT participants (30% to 34%, Figure 7A, left panel). As underlined in the previous section, 1-step- and 3-step games share the need to detect dominance properties (i.e., dominant or dominated actions) in the Own-payoffs matrix. Therefore, the successful acquisition of relevant resolution procedures (i.e., dominance detection) in 1-step games by High-CRT participants leads to beneficial transfer learning effects in 3-step games by triggering the formation of second-order beliefs.^8^

We also observe a *negative* phase effect in 3-step games in the C-2-step condition when comparing High-CRT and Low-CRT participants (*B* = −1.057, *z* = −2.30, *p* = 0.022). Indeed, in this context, High-CRT participants’ rate of Nash choices significantly decreases from 24% to 11% (Figure 7B, right panel). In comparison, Low-CRT participants’ sophistication remains stable (from 23% to 25%, Figure 7B, left panel). This suggests that learning procedures after feedback in 2-step games (C-2-step condition) had a detrimental effect on High-CRT participants’ choices in 3-step games, which require different (and more complex) resolution procedures and higher levels of strategic thinking.

Altogether, these results show that higher levels of cognitive reflection support the transfer of acquired knowledge to novel contexts (**Hypothesis 3c**). However, this transfer also emerges when the acquired knowledge is unsuitable for solving games in novel strategic scenarios, leading to maladaptive learning effects.

We also tested for working memory effects, running a model similar to Model 4 but using the WM-level and respective interactions in place of the CRT-level as a predictor (Model 5, see Table S8, Supplementary Information). Results did not reveal any interaction between WM-level and phase (*χ*2(1, *N* = 159) = 0.12, *p* = .729) and between WM-level, condition, and phase (*χ*2(2, *N* = 159) = 0.31, *p* = .855). Therefore, we can conclude that differences in working memory as measured by the N-Back task did not play a role in the increase in strategic sophistication in the Feedback treatment.

We also replicate the results on cognitive abilities in two additional models (Model 4b and Model 5b) in which we treat the cognitive level predictor as a continuous variable. All the main results are comparable with the main models (see Table S9 and Table S10, respectively).

### Information Acquisition Sequences (IASs)

Using process tracing analysis, we tested whether changes in information acquisition patterns reflected the observed learning effects. In particular, we hypothesize that learning by feedback in our different learning conditions is linked to the acquisition (in the Learning phase) of specific strategically relevant resolution procedures, i.e., specific Information Acquisition Sequences (IASs). We analyzed differences in the use of the three relevant IASs across phases, conditions, game classes, and cognitive abilities. We focused on data from the Feedback treatment since behavioral results did not show any learning effects in the Baseline treatment. Moreover, we used the cognitive reflection level as a predictor of interest for cognitive abilities, given that only cognitive reflection has been shown to modulate learning in behavioral analyses. We replicated the analyses separately for the three types of IASs in three mixed-effects logistic models (Models 6, 7, 8). The three models used trial-by-trial completion of the current IAS (0: IAS not completed; 1: IAS completed) as the binary dependent variable, condition (C-1-Step; C-2-step; C-3-step), game class (1-step; 2-step; 3-step), phase (Assessment; Reassessment), cognitive reflection level (High-CRT; Low-CRT) and their interactions as independent factors. We included a random effect on the intercept at the participant level. Results are reported in the Supplementary Information (Tables S11–S17).

For all the IASs, we observe a significant interaction between condition and phase, suggesting that the change in the use of specific information acquisition sequences depended on the learning context (IAS-1-step: *χ*2(2, *N* = 159) = 228.34, *p* < .001; IAS-2-step: *χ*2(2, *N* = 159) = 245.74, *p* < .001; IAS-3-step: *χ*2(2, *N* = 159) = 163.99, *p* < .001). Conversely, for none of the three IASs, we observe a significant three-way interaction between condition, phase, and game class (IAS-1-step: *χ*2(4, *N* = 159) = 1.25, *p* = .870; IAS-2-step: *χ*2(4, *N* = 159) = 1.38, *p* = .848; IAS-3-step: *χ*2(4, *N* = 159) = 0.78, *p* = .942).^9^ These results suggest that participants learned resolution strategies that depended on the learning context and that were not applied adaptively, i.e., depending on the game class, in the Reassessment phase. These results support **Hypothesis 4a**.

**Figure 8.**
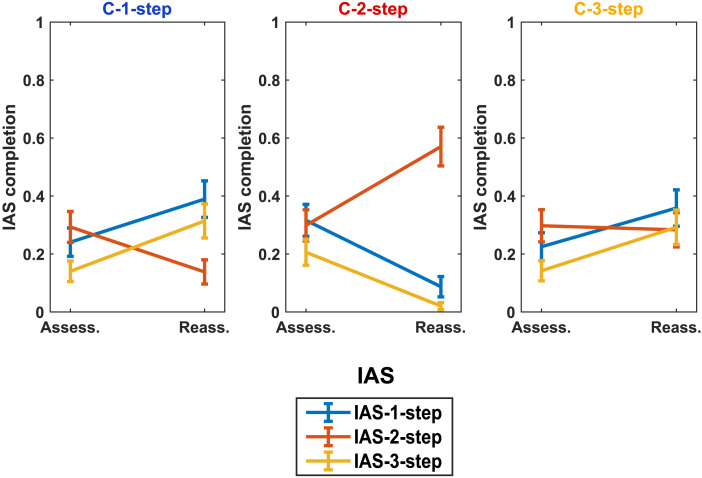
Line graphs of the proportion of games in which each IAS (IAS-1-step, IAS-2-step, IAS-3-step) was completed across phases (Assess.: Assessment; Reass.: Reassessment), in each of the three experimental conditions (C-1-step, C-2-step, C-3-step). Error bars represent between-subject standard errors of the mean. The color of the label of each condition identifies the IAS that is fundamental to solving the game class faced in the Learning phase.

Figure 8 reveals how the implementation of different IASs is modulated between the Assessment and the Reassessment phases across the three experimental conditions. In the C-1-step condition, where feedback in the Learning phase triggers the need for players to focus on the comparison between their own actions to detect dominance properties, we indeed observe an increase in the use of IAS-1-step (from 24% to 38%) and IAS-3-step (from 14% to 31%). Both these IASs are characterized by a thoughtful exploration of the participant’s own payoffs. In line with this interpretation, we observe a decrease (from 29% to 14%) in IAS-2-step, which is not required to solve the games faced in the Learning phase. On the contrary, results of the C-2-step condition show an increase (from 30% to 57%) in the use of IAS-2-step (necessary to solve the games faced in the Learning phase) and a parallel decrease in the use of IAS-1-step (from 31% to 9%) and IAS-3-step (from 20% to 2%). In the C-3-step condition, we observe an increase in the use of IAS-1-step (from 22% to 36%) and IAS-3-step (from 14% to 29%), in line with the acquisition of procedures needed to solve 3-step games (faced in the Learning phase). These results confirm that players in the Learning phase learn resolution procedures necessary in that specific environment and tend to re-apply them in similar and different contexts, in line with **Hypothesis 4a**.

The results of Model 6, 7, and 8 also support **Hypothesis 4b** revealing an interaction between cognitive reflection level and phase in modulating the use of the three IASs (IAS-1-step: *χ*2(1, *N* = 159) = 25.57, *p* < .001; IAS-2-step: *χ*2(1, *N* = 159) = 26.86, *p* < .001; IAS-3-step: *χ*2(1, *N* = 159) = 25.23, *p* < .001). Moreover, for all the three IASs we observe a significant three-way interaction between cognitive reflection level, condition and phase (IAS-1-step: *χ*2(2, *N* = 159) = 121.02, *p* < .001; IAS-2-step: *χ*2(2, *N* = 159) = 183.90, *p* < .001; IAS-3-step: *χ*2(2, *N* = 159) = 68.12, *p* < .001). Consistent with the reported absence of interaction effects between phase, condition, and game class, we did not observe any significant four-way interactions between phase, condition, game class, and cognitive reflection level.

**Figure 9.**
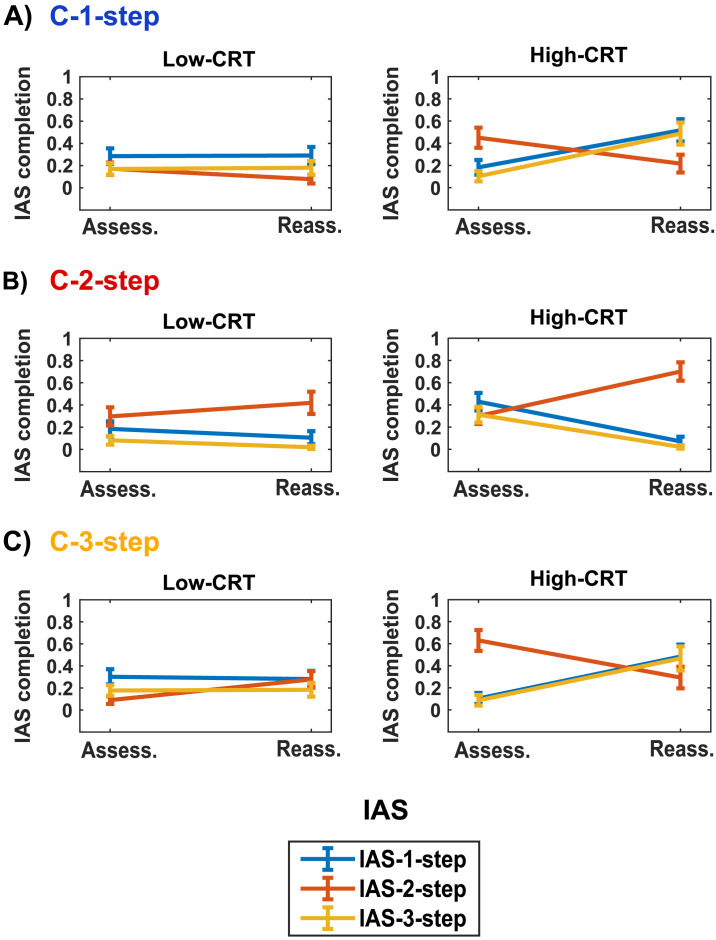
Line graphs of the proportion of games in which each IAS (IAS-1-step, IAS-2-step, IAS-3-step) was completed for High-CRT and Low-CRT participants, across phases (Assess.: Assessment; Reass.: Reassessment), in each of the three experimental conditions (A: C-1-step, B: C-2-step, C: C-3-step). Error bars represent between-subject standard errors of the mean. The color of the label of each condition identifies the IAS that is fundamental to solving the game class faced in the Learning phase.

Figure 9 and Tables S13, S15, and S17 (Supplementary Information) illustrate these results in detail. In the C-1-step condition (Figure 9A), we highlight that the observed increase (from the Assessment to the Reassessment phase) in the implementation of IAS-1-step and IAS-3-step is entirely guided by High-CRT participants (IAS-1-step: from 18% to 51%; IAS-3-step: from 10% to 48%). Conversely, Low-CRT participants do not modify significantly their rate of IAS-1-step (from 28% to 29%) and IAS-3-step (from 17% to 18%). Moreover, the decrease in the use of IAS-2-step is markedly higher in High-CRT participants (from 45% to 22%) than in Low-CRT participants (from 17% to 8%).

In the C-2-step condition (Figure 9B), High-CRT participants show a notable change in their information acquisition sequences: they increase their proportion of IAS-2-step (from 30% to 70%) and decrease their rate of IAS-1-step (from 43% to 7%) and IAS-3-step (from 31% to 2%). On the contrary, information acquisition sequences remain more stable in Low-CRT participants (IAS-1-step: from 17% to 10%; IAS-2-step: from 30% to 42%; IAS-3-step: from 7% to 2%).

In the C-3-step condition (Figure 9C), High-CRT participants increase significantly their proportion of IAS-1-step (from 10% to 48%) and IAS-3-step (from 9% to 47%), whereas Low-CRT participants do not (IAS-1-step: from 30% to 28%; IAS-3-step: from 18% to 18%). Interestingly, we observe an opposite pattern concerning IAS-2-step: High-CRT participants decrease the use of this information acquisition sequence (from 63% to 29%), whereas Low-CRT participants increase the use of IAS-2-step (from 9% to 28%).

These last findings and behavioral results also support **Hypothesis 3d**, suggesting that the cognitive reflection level determined the resolution procedure learned in the Learning phase and transferred in the Reassessment phase. High-CRT participants learned procedures appropriate for the implementation of three steps of strategic thinking, which enabled them to improve their sophistication in 3-step games; on the contrary, Low-CRT participants acquired procedures entailing two steps of reasoning, which led to an increase in Nash choices in 2-step games only, as highlighted in the behavioral results.

## DISCUSSION

### Summary and Significance

Strategic sophistication refers to anticipating others’ behavior and responding with appropriate actions. Recent evidence suggests that strategic thinking may be enhanced by feedback or training (Gill & Prowse, 2016; Knoepfle et al., 2009; Marchiori et al., 2021; Selten et al., 2003; Verbrugge et al., 2018; Zonca et al., 2019, 2021). Nonetheless, research on learning in strategic interaction is still in its infancy. No studies have explored how exogenous factors (e.g., the learning environment) interact with endogenous factors (e.g., individual cognitive abilities) to increase strategic sophistication. To address this gap, we designed an experiment that manipulated the learning context and measured learning success and cognitive abilities. We aimed to test whether improvements in strategic interaction depend on the interplay between the learning context and individual cognitive abilities.

Choice and process tracing results reveal that this is indeed the case. The learning environment significantly influences what participants learn and, consequently, whether and under which circumstances they increase their strategic sophistication in future interactions. Feedback in a specific strategic environment improves players’ optimality in similar environments, even when feedback is later removed. This learning effect is more pronounced in players with higher levels of cognitive reflection. Moreover, higher cognitive reflection enables learning in complex strategic scenarios requiring three steps of strategic thinking, revealing a critical cognitive boundary for acquiring highly sophisticated strategies. Some individuals, particularly those with higher cognitive reflection, can transfer acquired knowledge to new strategic contexts. However, this transfer is a double-edged sword, as it is not implemented in an adaptive, context-dependent manner. When facing a novel environment, even players with high cognitive reflection show a behavioral advantage only if the learned strategies suit that specific environment. Otherwise, they fail to adapt, resulting in suboptimal choices.

### Learning Mechanisms

Our results show that learning from feedback in matrix games emerges through acquiring more effective resolution procedures rather than developing a general, flexible increase in strategic sophistication. Process tracking results reveal that the transfer of novel patterns of information acquisition is strongly dependent on the learning context and is re-applied independently of the payoff structure of the novel environment. The employment of non-adaptive learning mechanisms that privilege the systematic re-application of resolution procedures recalls several lines of research in different fields, which introduced the distinctions between problem-model and direct-translation strategies in problem-solving (Boote & Boote, 2018; Mayer & Hegarty, 1996), model-based and model-free learning (Daw et al., 2005, 2011; Konovalov & Krajbich, 2016), and rule abstraction and memorization in category learning (McDaniel et al., 2014). Moreover, our findings remind classical studies on problem-solving, highlighting the emergence of mechanization of resolution procedures due to previous exposure to similar problems (Bilalić et al., 2008; Blech et al., 2020; Cherubini & Mazzocco, 2004; Luchins, 1942, Luchins & Luchins, 1987).

Our results suggest that learning resolution procedures represent a primary form of learning through feedback in strategic interaction. Feedback allows a clearer understanding of the opponent’s behavior, triggering the exploration and application of new strategies. However, simple feedback exposure from a single class of games is not enough to develop a flexible adaptation of sophistication in heterogeneous spaces of strategic environments, since learned resolution strategies are applied rigidly across different contexts. Depending on the current payoff structure and the potential sophistication of the opponent, this may lead to maladaptive transfer effects (Hedden & Zhang, 2002; Verbrugge et al., 2018).

### The Role of Inter-Individual Differences in Cognitive Abilities

To explore the role of inter-individual differences in cognitive abilities in predicting strategic learning through feedback, we assessed two cognitive measures: the Cognitive Reflection Test and the N-back task. Consistent with previous literature, we found that both tests predict higher levels of strategic sophistication, specifically a greater reliance on strategies entailing up to two steps of strategic thinking in the absence of feedback. This suggests that cognitive abilities, as measured by the CRT and N-back task, support strategic thinking. However, higher cognitive reflection and working memory skills alone are insufficient to increase reliance on more complex strategies involving three steps of strategic thinking and second-order beliefs without feedback. Even individuals with high cognitive skills require support to elevate their strategic sophistication to a level requiring three steps of strategic thinking (Costa-Gomes & Weizsäcker, 2008; Polonio & Coricelli, 2019; Zonca et al., 2020a, 2021).

After feedback exposure, the two cognitive measures predict behavior in distinct ways. The N-back score does not predict sophistication enhancement, implying that higher working memory does not necessarily aid in acquiring new strategic knowledge, nor do lower working memory skills hinder learning and applying new strategies. In contrast, the CRT score predicts the adoption of novel and effective strategies following feedback exposure. We propose that higher levels of cognitive reflection enhance understanding of the strategic environment and the opponent’s behavior and facilitate the exploration of alternative strategies to improve performance. Previous studies (Devetag & Warglien, 2008; Mazzocco et al., 2013) have highlighted the crucial role of information acquisition, integration, and representation mechanisms in determining strategic sophistication in games. Specifically, the CRT score has been linked to better integration and representation of relevant information in a game matrix, which correlates with higher strategic sophistication (Zonca et al., 2020b). Our results further emphasize that high levels of cognitive reflection are necessary for acquiring, applying, and transferring complex procedures to navigate intricate game structures. This suggests that some game structures may be too complex for many players, underscoring the importance of bounded rationality in modeling strategic sophistication (Alaoui & Penta, 2016; Frydman & Nunnari, 2021; Jin, 2021).

Overall, feedback is an effective tool for enhancing strategic sophistication, particularly when coupled with high cognitive reflection. We suggest that players, even the most sophisticated ones, initially approach matrix games with the assumption that their opponent has a low level of sophistication (Hedden & Zhang, 2002). Feedback exposure can prompt players with high cognitive reflection to explore the opponent’s incentives and beliefs more thoughtfully, leading to the development of second-order beliefs and higher-order reasoning. The role of cognitive reflection in modulating the ability to elevate strategic thinking in a given environment may explain why a significant proportion of less sophisticated players fail to increase their sophistication, even when informed they are playing against highly sophisticated individuals (Agranov et al., 2012; Alaoui et al., 2020; Alaoui & Penta, 2022; Jin, 2021).

This study enriches our understanding of the relationship between cognitive abilities and strategic sophistication by demonstrating that higher cognitive reflection offers greater learning opportunities, while lower working memory abilities do not pose a barrier to enhancing strategic sophistication.

### Theoretical Models of Strategic Sophistication

Our results provide novel insights into the empirical and theoretical validity of recent models of strategic behavior (Alaoui & Penta, 2016; Frydman & Nunnari, 2021), reinforcing a view of strategic sophistication that is both 1) modulated by the available knowledge of the current strategic environment and 2) constrained by cognitive limitations. Specifically, our findings show that players can learn to implement new strategies and elevate their level of strategic thinking after receiving feedback. However, cognitive constraints can limit the effective transfer of acquired knowledge to different strategic settings and hinder the development of high-level (e.g., third-order) strategic reasoning.

In Alaoui and Penta’s model of strategic sophistication (2016, 2022), the depth of strategic reasoning in games is determined endogenously through a cost-benefit analysis, which considers inter-individual differences in cognitive abilities (i.e., the cost of reasoning). According to this model, boundedly rational players exhibit less sophisticated behavior because their incentives to engage in deeper thinking (i.e., the current payoffs) are outweighed by the relative cognitive cost. This idea aligns with the observed correlation between individual CRT scores and strategic sophistication: individuals with lower CRT scores tend to rely on intuitive thinking because their cognitive costs are higher. Our findings can be interpreted within this theoretical framework. When exposed to feedback, individuals with higher cognitive reflection—due to lower cognitive costs—are better able to extract information about their opponent’s behavior and enhance their strategic sophistication. Conversely, individuals with lower cognitive reflection struggle to deepen their reasoning, particularly in more complex games that demand substantial cognitive resources.

Our findings can also be interpreted through the lens of Frydman and Nunnari’s recent model of strategic sophistication (2021), which introduces the concept of cognitive noise. Cognitive noise disrupts strategic thinking by distorting the encoding and representation of matrix payoffs. First, our results suggest that bounded rationality influences the early stages of game resolution, particularly during the selection and acquisition of relevant information (e.g., payoffs, actions, dominance properties). Even after feedback exposure, players with low cognitive reflection appear unable to effectively explore the game matrix to identify strategically relevant properties within a specific context. Second, and most importantly, feedback exposure may reduce the influence of cognitive noise by providing clues about the opponent’s strategy, thereby narrowing the perceived action space. This reduction in cognitive noise can lead to enhanced strategic sophistication, especially for players less affected by cognitive noise—namely, those with higher levels of cognitive reflection.

### Methodological Considerations

The present study leverages mouse tracking to disclose the type of resolution procedures learned by participants through feedback. Other techniques, including eye-tracking and reaction time analysis, can be used to understand better how decision-making in strategic contexts unfolds. These techniques carry their advantages and disadvantages (see Stillman et al., 2018). Eye-tracking provides a unique window into the dynamics of the visual attentional focus during task exploration, but it is relatively costly in terms of equipment, knowledge, and experimental acquisition time. Reaction times are extremely easy to collect and powerful in characterizing specific cognitive processes (e.g., intuitive vs. deliberative thinking; Rubinstein, 2007), but they are opaque in identifying other types of mechanisms. For instance, in the context of the current study, reaction times represent an ambiguous indicator of sophistication and learning: shorter reaction times may represent a less thoughtful exploration of the available information and the emergence of unsophisticated behavior but also reveal the acquisition and systematic implementation of an efficient and effective resolution strategy. Mouse-tracking may offer a good compromise between costs and benefits. As shown in the present study, the analysis of mouse-click patterns can be extremely powerful in revealing goal-directed processes, despite its cheapness and ease of implementation.

### Future Directions

The present study shows that learning by feedback can enhance strategic sophistication, but this is limited by 1) individual cognitive abilities and 2) exposure to homogenous learning contexts.

Participants with low cognitive reflection might need more explicit and extended training to implement second-order beliefs and higher-order steps of strategic thinking. In this regard, results from research on false belief tasks suggest that learning by feedback with explicit explanation can enhance second-order (Arslan et al., 2020) and first-order (Appleton & Reddy, 1996; Clements et al., 2000; Kloo & Perner, 2008; Melot & Angeard, 2003) Theory of Mind reasoning. Another possible path for enhancing higher-order levels of strategic thinking in matrix games is using sequential games (see Hedden & Zhang, 2002; Zhang et al., 2012) as training. Sequential games highlight the recursive nature of strategic thinking and may trigger the implementation of second-order beliefs in complex matrix games, as used in the present study. This kind of training may be more effective if training with sequential games is offered stepwise (Verbrugge et al., 2018) by sequentially increasing game complexity.

Participants with high cognitive reflection may fail to adapt to different strategic contexts when learning happens in one context only. Future studies may offer a more heterogeneous learning environment (e.g., including games with different payoff structures) to investigate whether we can elicit a more flexible and generalized enhancement of strategic sophistication.

Our design does not provide evidence of the persistence of the training effects over time. Further studies should investigate the persistence of learning outcomes over time by adding strategic sophistication reassessments at medium and long-term periods.

Eventually, future studies may enrich the investigation of the role of inter-individual variability in modulating learning in strategic interaction by using other types of cognitive measures (e.g., measuring different types of working memory or cognitive skills).

Our study started to scratch the surface by showing that learning by feedback depends on the interplay between the learning environment and individual cognitive abilities; however, further research is needed to draw an exhaustive picture of learning in strategic interaction, involving different types of learning manipulation and targeting other types of individual differences to disclose exhaustively the sources of behavioral heterogeneity.

### Concluding Remarks

The present study showed that the learning environment interacts with individual cognitive abilities to shape what individuals learn and implement in strategic interactions. These findings should be considered when developing interventions (e.g., training, nudging) to enhance strategic sophistication in research and application contexts. The twofold impact of the learning context should be considered if we seek to improve proper strategic sophistication (i.e., the ability to reason deeply and exhaustively about all actors’ decisions in a strategic context) rather than mere acquisition and reapplication of resolution procedures.

Strategic sophistication is a pervasive ability in our everyday life. Our results provide crucial evidence to fuel further research on strategic sophistication enhancement and to support application in relevant educational and professional settings.

## ACKNOWLEDGMENTS

We thank the editor Maarten Speekenbrink and the anonymous reviewers for their feedback on our work.

## FUNDING INFORMATION

This research was supported by internal funds from the Department of Psychology at the University of Milano-Bicocca.

## AUTHOR CONTRIBUTIONS

Conceptualization: L.P., C.R., A.R.; Data curation: J.Z.; Formal analysis: J.Z.; Funding acquisition: C.R.; Investigation: L.D.M.; Methodology: L.P., C.R.; Project administration: L.P., C.R., J.Z.; Software: J.Z., C.R., L.P., L.D.M.; Visualization: J.Z., L.D.M.; Writing – original draft: J.Z., L.D.M.; Writing – review & editing: J.Z., C.R., L.P., L.D.M.

## DATA AVAILABILITY STATEMENT

Datasets and materials supporting the analyses and figures included in the current study, in addition to supplementary files describing methods and experimental stimuli in detail, are available in a dedicated OSF repository: https://osf.io/5pmhs/?view_only=b52d127779e84a39aea45f8927043f24.

## Notes

^1^ Nash equilibrium refers to a situation in which no player is incentivized to change his strategy unilaterally, given the strategy chosen by the other player. It is a joint action solution, where no individual agent can improve his outcome by altering his strategy profile while the other agent keeps his strategy unchanged.^2^ For a full list of games of the Assessment phase, see Supplementary Information, Supplementary Methods, Figure S1. The games of Learning and Reassessment phases have the same payoff structure but slightly different payoffs. For the complete list of the 75 games, see the dedicated OSF repository: https://osf.io/5pmhs/?view_only=b52d127779e84a39aea45f8927043f24.^3^ For an English translation of the participants’ instructions, see the Supplementary Information (Supplementary Methods, Participants’ instructions). Instructions are also included in a the dedicated OSF repository: https://osf.io/5pmhs/?view_only=b52d127779e84a39aea45f8927043f24.^4^ To watch a video example of a typical trial, see the dedicated OSF repository: https://osf.io/5pmhs/?view_only=b52d127779e84a39aea45f8927043f24.^5^ Videos with examples of each of the three IASs can be found in the dedicated OSF repository: https://osf.io/5pmhs/?view_only=b52d127779e84a39aea45f8927043f24.^6^ Excluded participants were distributed across conditions as follows. Feedback treatment: *N* = 10 (*N* = 2 for C-1-step condition; *N* = 4 for C-2-step condition; *N* = 4 for C-3-step condition). Baseline treatment: *N* = 4 (*N* = 1 for C-1-step condition; *N* = 2 for C-2-step condition; *N* = 1 for C-3-step condition).^7^ Descriptive statistics of our cognitive measures (in all treatments and conditions, excluding participants who do not meet the inclusion criteria of the main analyses). CRT score: Mean = 3.47, *SD* = 1.86. 2-back: Mean = 0.68, *SD* = 0.17. 3-back: Mean = 0.60, *SD* = 0.17. N-back score: Mean = 0.64, *SD* = 0.16.^8^ We also tested whether learning in 3-steps games in C-1-step and C-3-step conditions in the Feedback treatment could be simply explained by a decrease in the rate of selection of the dominated action, which might have increased participants’ general rate of Nash choices in the Reassessment phase. We compared the proportion of games in which High-CRT participants chose the dominated action between the Reassessment and Assessment phases in both C-1-step and C-3-step conditions. Results show that the rate of dominated choices did not decrease across phases, excluding this interpretation of the results. In fact, participants rarely chose the dominated action already in the Assessment phase (C-1-step. Assessment: 11%; Reassessment: 7%. Wilcoxon signed-rank test, *z* = −0.504, *p* = 0.614. C-3-step. Assessment: 9%; Reassessment: 11%. Wilcoxon signed-rank test, *z* = −0.504, *p* = 0.614).^9^ See full results in the Supplementary Information (Tables S13, S15, and S17).

## Supplementary Material


