# Supplementary material for: Learning in Interactive Decision-Making: The Interplay Between Cognitive Abilities and the Strategic Environment
Source: Open Mind (Camb). 2025 Jan 23;9:210–39. doi: 10.1162/opmi_a_00186 (PMC11793201; doi:10.1162/opmi_a_00186)
Supplement: Supplementary file 1 [file opmi-09-210-s001.docx]

**Supplementary Information**

**Supplementary Methods**

**
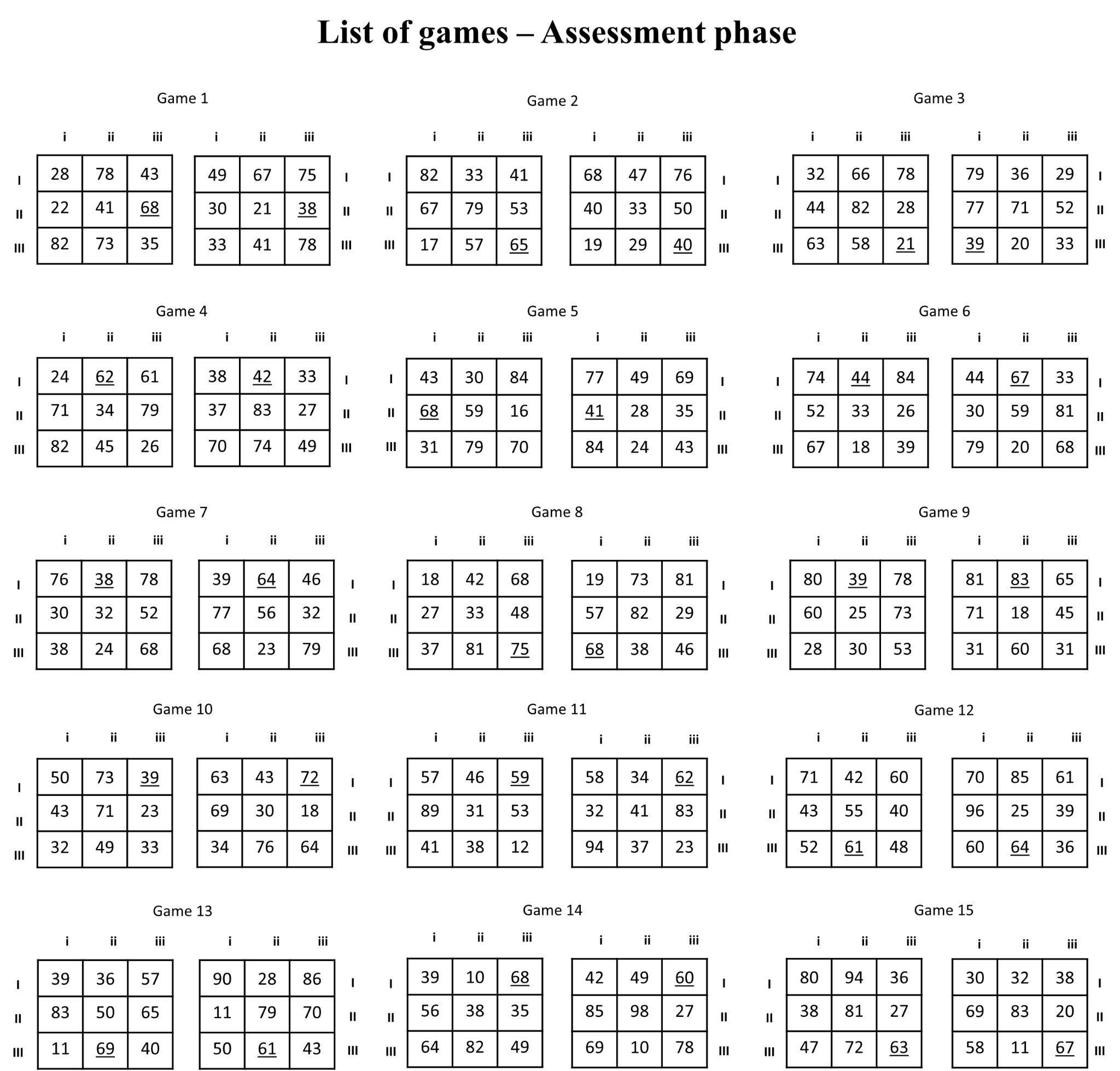
**

**Figure S1. List of the 15 games of the Assessment phase.** The games of Learning and Reassessment phases have the same payoff structure but slightly modified payoffs. For a complete list of all the 75 games, see the dedicated repository: https://osf.io/5pmhs/?view_only=b52d127779e84a39aea45f8927043f24

**Cognitive measures**

**Cognitive Reflection Test – long (CRT-L) version**

Here we report the six items and relative correct answers of the CRT-L employed in the experiment. CRT score is computed by summing the number of correct responses.

1) A piece of chocolate and a piece of candy cost €1.10 in total. The piece of chocolate costs €1 more than the piece of candy. How much does the piece of candy cost? … cents (Correct answer: 5)

2) If it takes 5 machines 5 minutes to make 5 widgets, how long would it take 100 machines to make 100 widgets? … minutes (Correct answer: 5)

3) There is a patch of lily pads in a lake. Every day, the patch doubles in size. If it takes 48 days for the patch to cover the entire lake, how long would it take for the patch to cover half of the lake? … days (Correct answer: 47)

4) If 3 store clerks can wrap 3 toys in 1 hour, how many store clerks would be needed to wrap 6 toys in 2 hours? … clerks (Correct answer: 3)

5) Marco has a grade that is both the 15th highest and the 15th lowest grade in his class. How many students are in Marco’s class? … students (Correct answer: 29)

6) In a team of athletes, the tall athletes jointly win 3 times as many medals as the short athletes. Over the past year, the team has won 60 medals. How many medals were won by short athletes? … medals (Correct answer: 15)

**Participants’ instructions**

**Main task**

**Introduction (All treatments and conditions)**

Welcome. You will be presented with three experiments. The reward in Euros that you will receive at the end of the experiment will depend on the score you achieve in the first and second experiments.

You will have as much time as you need to complete the experiment. When you finish, please wait silently until the experimenter calls you for payment. Keep in mind that you may need to wait for other participants to finish the experiment as well.

**The experiment (All treatments and conditions)**

In the first experiment, you will have to make a series of interactive decisions with the goal of obtaining as many points as possible. The term 'interactive' means that the outcome of your decision will depend on the combination of your choice and the choice of the counterpart, which will be a computer throughout the experiment. In each trial, you and the computer will decide simultaneously, so neither you nor the computer will have information about the choice made by the respective counterpart at the time you make your decision.

The computer will play with the aim of earning as many points as possible and will follow the same decisional rule throughout the experiment. Therefore, its strategy will not change, regardless of your choices. The structure of each choice problem, which we will now call a 'GAME,' will be represented in the form of two tables, presented one next to each other. The table displayed on the left contains all your potential payoffs for this game (blue), while the table displayed on the right contains all the potential payoffs for the computer (red).

As you can see, a game consists of 3 rows ('I,' 'II,' 'III') and 3 columns ('i,' 'ii,' 'iii'). You will always cover the role of ROW PLAYER, while the computer will always cover the role of COLUMN PLAYER. Consequently, your possible choices are represented by the rows in both tables ('I' row at the top, 'II' row in the middle, and 'III' row at the bottom), while the computer's choices are represented by the columns in both tables ('i' column on the left, 'ii' column in the middle, and 'iii' column on the right).


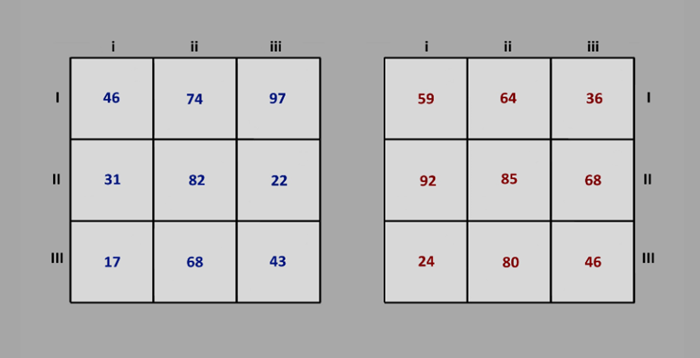


The combination of the row selected by you and the column selected by the computer results in a cell within the left table and a cell within the right table. The cell on the left expresses your payoff for that game, while the cell on the right table expresses the computer's payoff. For example, referring to the game shown below, if you were to choose the 'II' row and the computer were to choose the 'i' column, your score would be that reported in the cell resulting from the intersection of the selected row and column within the left table, while the computer's score would be that reported in the cell resulting from the intersection of the selected row and column within the right table. Consequently, in this example, you would obtain 31 points, while the computer would obtain 92.


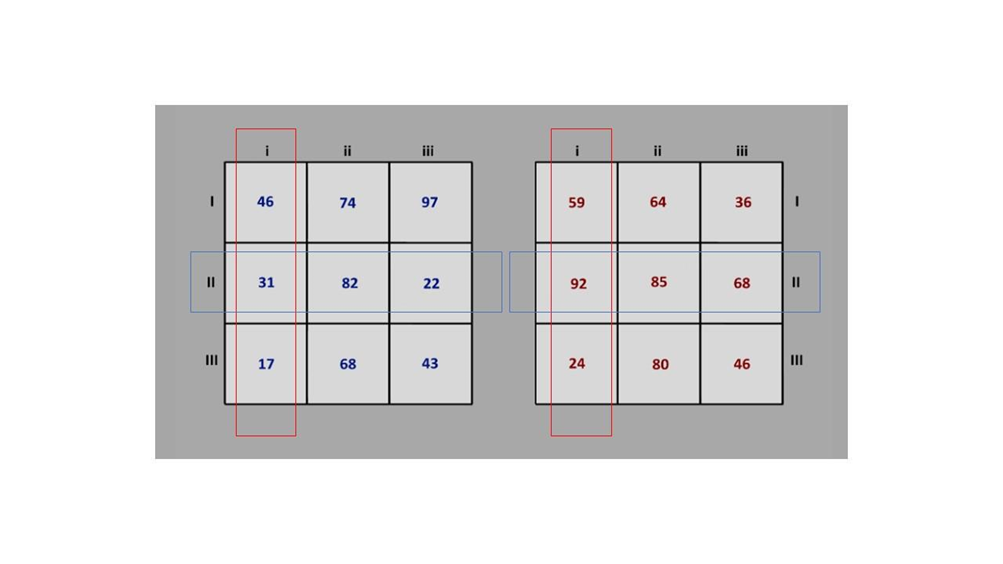


Remember that you cannot directly choose one of the cells in the tables, but only one of the rows, while the computer can only choose one of the columns. Only the combination of both choices will result in the selection of a cell in both tables, corresponding to your and the computer's payoffs. Neither you nor the computer will have information about the choice made by the respective counterpart at the time you are making your decision.

The experiment will consist of 3 phases, in each of which you will be presented with 15 games, corresponding to 15 different interactive decisions, each independent of the others. In each table, all payoffs will always be covered by colored boxes, as shown in the figure below. The boxes covering your payoffs will be blue, while those covering the computer's payoffs will be red. You will have the opportunity to 'open' these boxes to view the payoffs as many times as you want. However, you can open a maximum of 6 boxes simultaneously.


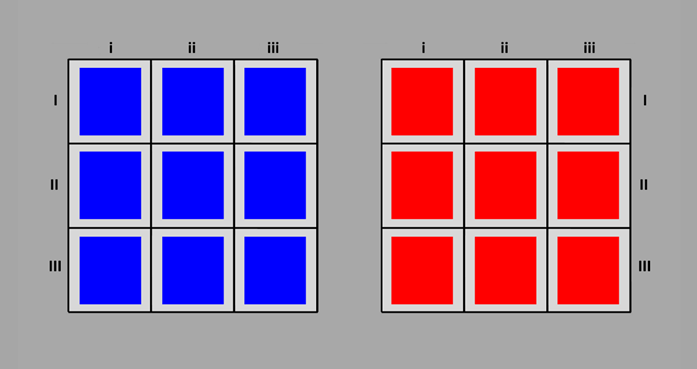


To select the boxes you would like to open, you simply need to click on them using the left mouse button. To select more than one at a time, you can click (left mouse button) on multiple boxes, or you can hold down the left mouse button and move the cursor over the boxes you are interested in. The selected boxes will be shaded in a lighter shade of blue or red.


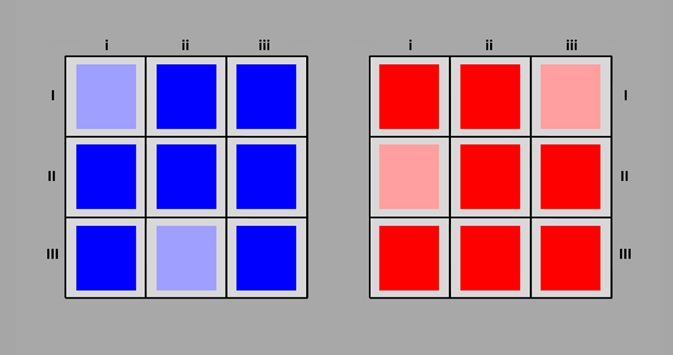


To cancel a potentially incorrect selection, simply click/select the same boxes again, and they will return to the deselected state. To open the selected boxes, click once with the right mouse button anywhere on the screen. Before making a new selection, you must first close any still-open boxes by clicking the right mouse button again.


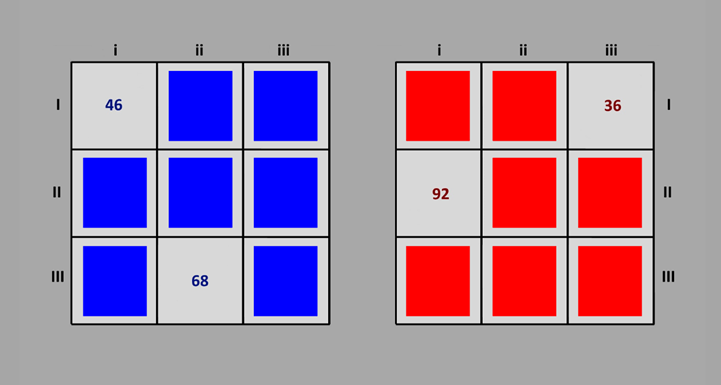


**Assessment phase (All treatments and conditions)**

In Phase 1 of the choice experiment, you will be presented with 15 games, corresponding to 15 different interactive choices, each independent of the others. To make your decision in a game, simply press the '1' key on your keyboard if you want to select row 'I,' the '2' key if you want to select row 'II,' and the '3' key if you want to select row 'III.' For each game, once you have made your decision, you will move directly to the next game without receiving any information about the computer's choice and the respective payoffs obtained by you and the computer. This means that, in Phase 1, you will not know the outcome of your choices. Remember that the computer will play with the aim of earning as many points as possible and will follow the same decision rule throughout the experiment.

**Learning phase (Feedback treatment, all conditions)**

In Phase 2, you will be presented with another set of 15 games, and you will once again make your choices by interacting with the computer. The decision rule used by the computer in Phase 2 will be the same as that used in Phase 1. However, in this phase, after you make your choice, you will also be informed about the computer's choice and the outcome of the game. The image below illustrates how this information will be presented to you. In this example, you chose the row "III," and the computer chose the column "ii." As a result, you gained 68 points, and the computer gained 80 points. Keep in mind that this is just a demonstrative example and does not reveal the computer's decision rule. The rule used by the computer will remain consistent throughout all three phases of the experiment and aims to maximize its point accumulation.


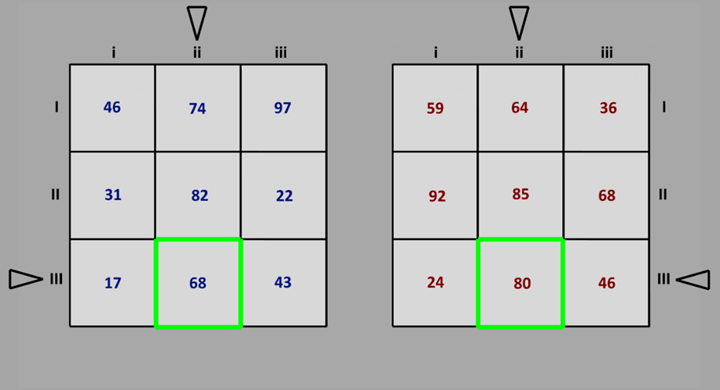


**Learning phase (Baseline treatment, all conditions)**

In Phase 2, you will be presented with another 15 games, and you will again make your choices by interacting with the computer. The decision rule used by the computer in Phase 2 will be the same as that used in Phase 1.

**Reassessment phase (All treatments, all conditions)**

In Phase 3, you will be presented with another 15 games, corresponding to 15 different independent interactive choices. The decision rule assumed by the computer will be the same as that used during Phases 1 and 2. In this phase, as in the first and second phase, no additional information will be provided about the computer's choice or the results obtained by you and the computer.

**Payment and concluding remarks**

The reward you will receive for the first experiment will be calculated as follows: Three games will be randomly selected, one for each of the three phases. The points you earn in the three games will be summed. 10 points will be paid as 0.70 Euros. For example, if the sum of points in the first experiment is 180, you will earn 11.20 Euros (18 X 0.7 = 12.60). Before the start of the experiment, you will be asked to answer a simple questionnaire to verify that you have understood the instructions. If you feel uncertain about anything during the questionnaire, you can return to the beginning of the instructions by pressing the space bar. At the end of the questionnaire, you will be given the opportunity to play two practice trials to become familiar with the graphical interface of the experiment and the procedure for selecting/opening cells. If you have any other doubts or questions, feel free to ask for clarification from the experimenter at any time.

**Cognitive measures**

**2-back task**

In this experiment, you will be presented with a series of letters that will appear one by one on the screen. Each letter will be displayed for one second at the center of the screen, then it will be replaced by a gray screen for one second, after which another letter will appear, and so on. Every time a letter appears on the screen, you will need to indicate whether the same letter appeared 2 letters ago. The letters may appear in uppercase or lowercase, but the only thing that matters is the identity of the letter. So, if 'A' appears, then after two seconds 'n' appears, and after another two seconds 'a' appears, the correct response is that 'a' appeared two letters ago, even if the first time it appeared in uppercase. Each time a letter is displayed on the screen, you must respond YES if the letter did indeed appear two letters ago, and NO if a different letter appeared two letters ago.

To respond to each new letter, you will use the 'S' key to answer 'Yes, the same letter appeared two letters ago.' Or the 'L' key to answer 'No, this letter did not appear two letters ago.' Before the start of the experiment, there will be a training session where you will be shown whether the response is correct or not. Make sure you understand the examples before starting the training session.

The training session will begin shortly. Remember: Every time a letter appears, press the 'S' key if the same letter was presented 2 letters ago (both in lowercase and uppercase), and the 'L' key if the letter presented earlier was not presented two letters ago. Stay focused: during the actual experiment, no feedback will be provided. Do not press any key when the very first two letters are presented, as obviously, there won't have been enough letters preceding them for you to make a decision. During the actual experiment, you will not receive any feedback. Your earnings (in Euros) will depend on your accuracy in the experiment. In particular, you will earn 0.02 Euro for each correct response in each of the 100 trials. If you respond correctly to every trials, you can earn a total of 2 Euros. Tips: Stay focused on the task, don't worry too much if you miss a letter, but rather try to get back on track, and remember which key corresponds to which response (the 'S' key, the 'L' key).

**3-back task**

This time, you will be asked to indicate whether the currently displayed letter is the same letter displayed 3 LETTERS AGO. The other rules remain the same: Every time a letter appears, press the 'S' key if the same letter was presented 3 letters ago (both in lowercase and uppercase), and the 'L' key if the letter presented before was not presented before. During the experiment, you will not receive any feedback. Your earnings (in Euros) will depend on your accuracy in the experiment. In particular, you will earn 0.02 Euro for each correct response in each of the 100 trials. If you respond correctly to every trials, you can earn a total of 2 Euros.

**Cognitive Reflection Test (CRT)**

The questionnaire is about to begin. Read the following questions carefully and type your response using the keyboard; it will appear directly in the box below.

**Supplementary Results**

Descriptive statistics

| **Treatment** | **Condition** | **Game class** | **Assessment** | **Reassessment** |
| --- | --- | --- | --- | --- |
| Feedback | C-1-Step | 1-step | 0.74 (0.32) | 0.71 (0.28) |
|  |  | 2-step | 0.58 (0.38) | 0.53 (0.37) |
|  |  | 3-step | 0.26 (0.27) | 0.46 (0.32) |
|  | C-2-step | 1-step | 0.72 (0.28) | 0.72 (0.30) |
|  |  | 2-step | 0.63 (0.34) | 0.80 (0.34) |
|  |  | 3-step | 0.24 (0.21) | 0.18 (0.24) |
|  | C-3-step | 1-step | 0.75 (0.29) | 0.69 (0.36) |
|  |  | 2-step | 0.52 (0.36) | 0.69 (0.36) |
|  |  | 3-step | 0.22 (0.26) | 0.50 (0.38) |
|  | All | All | 0.52 (0.17) | 0.59 (0.21) |
| Baseline | C-1-Step | 1-step | 0.77 (0.23) | 0.78 (0.25) |
|  |  | 2-step | 0.60 (0.33) | 0.39 (0.36) |
|  |  | 3-step | 0.21 (0.15) | 0.14 (0.22) |
|  | C-2-step | 1-step | 0.84 (0.17) | 0.86 (0.16) |
|  |  | 2-step | 0.63 (0.33) | 0.71 (0.31) |
|  |  | 3-step | 0.12 (0.18) | 0.18 (0.24) |
|  | C-3-step | 1-step | 0.82 (0.27) | 0.82 (0.25) |
|  |  | 2-step | 0.58 (0.37) | 0.55 (0.36) |
|  |  | 3-step | 0.24 (0.17) | 0.24 (0.24) |
|  | All | All | 0.53 (0.16) | 0.52 (0.15) |

**Table S1.** Descriptive statistics reporting the average proportion of Nash equilibrium choices (between-subject standard deviations in brackets) by treatment (Feedback; Baseline), condition (C-1-step; C-2-step; C-3-step), game class (1-step; 2-step; 3-step) and phase (Reassessment; Assessment).


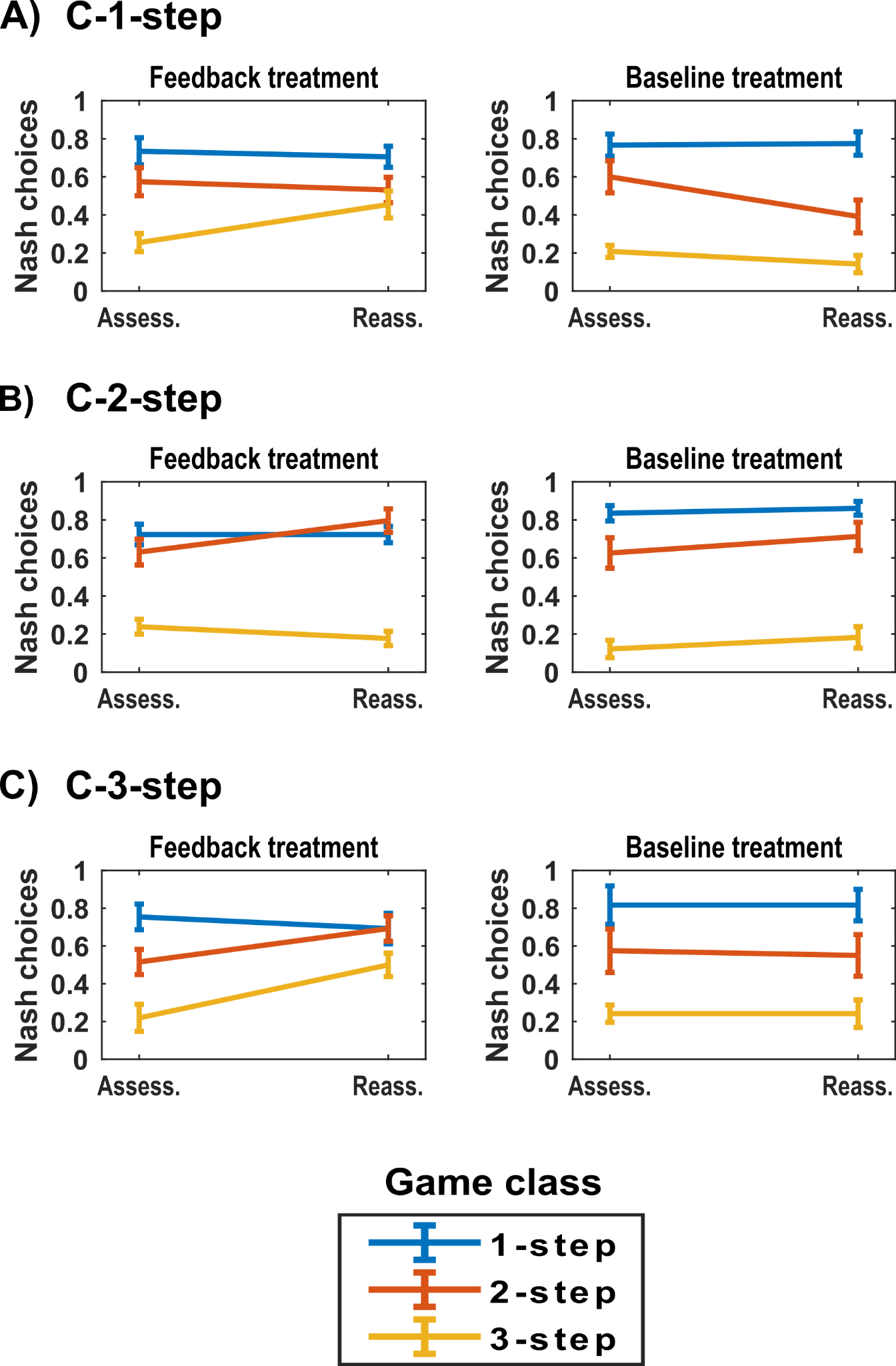


**Figure S2. Interplay between the presence of feedback, the learning context and the strategic environment in modulating learning.** Proportion of Nash choices across treatments (Feedback; Baseline), phases (Assess.: Assessment; Reass.: Reassessment), game classes (1-step, 2-step, 3-step games) in each of the three experimental conditions (**A**: C-1-step, **B**: C-2-step, **C**: C-3-step). Error bars represent between-subject standard errors of the mean. The color of the label of each condition identifies the game class faced in the Learning phase. Thus, in C-1-step (blue label), players play 1-step games (blue lines), in C-2-step (red label), players play 2-step games (red lines), in C-3-step (gold label), players play 3-step games (gold lines). Combinations of conditions and game class with matched colors (e.g., C-1-step condition and 1-step-games, in blue) highlight context-specific learning effects, whereas combinations with unmatched colors express transfer learning effects.

**Model 1**

Omnibus results:

| Nash choice | df | chi2 | p |
| --- | --- | --- | --- |
| Treatment | 1 | 1.08 | 0.300 |
| Condition | 2 | 2.82 | 0.245 |
| Treatment*Condition | 2 | 1.86 | 0.394 |
| Game class | 2 | .961.31 | < 0.001 |
| Treatment*Game class | 2 | 52.50 | < 0.001 |
| Condition*Game class | 4 | 53.29 | < 0.001 |
| Treatment*Condition*Game class | 4 | 3.21 | 0.524 |
| Phase | 1 | 5.82 | 0.016 |
| Treatment*Phase | 1 | 8.64 | 0.003 |
| Condition*Phase | 2 | 9.60 | 0.008 |
| Treatment*Condition*Phase | 2 | 10.15 | 0.006 |
| Game class*Phase | 2 | 5.40 | 0.067 |
| Treatment*Game class*Phase | 2 | 11.77 | 0.003 |
| Condition*Game class*Phase | 4 | 20.80 | < 0.001 |
| Treatment*Condition*Game class*Phase | 4 | 13.05 | 0.011 |
| N. obs | 6900 |  |  |
| N. groups | 230 |  |  |

**Table S2**. Omnibus results of Model 1.

Simple effects: effect of Phase

| Nash choice (Reass.-Assess.) | B | Std. Err. | z | p | 95% Conf. Inter. | |
| --- | --- | --- | --- | --- | --- | --- |
| Feedback treatment |  |  |  |  |  |  |
| C-1-step condition |  |  |  |  |  |  |
| 1-step games | - 0.157 | 0.198 | - 0.79 | 0.428 | - 0.546 | 0.232 |
| 2-step games | - 0.197 | 0.1811 | - 1.09 | 0.277 | - 0.553 | 0.158 |
| 3-step games | 1.000 | 0.195 | 5.13 | < 0.001 | 0.618 | 1.382 |
| C-2-step condition |  |  |  |  |  |  |
| 1-step games | - 0.000 | 0.204 | - 0.00 | 1.000 | - 0.399 | 0.399 |
| 2-step games | 0.893 | 0.208 | 4.28 | < 0.001 | 0.484 | 1.301 |
| 3-step games | - 0.398 | 0.224 | - 1.77 | 0.076 | - 0.838 | 0.042 |
| C-3-step condition |  |  |  |  |  |  |
| 1-step games | - 0.341 | 0.207 | - 1.65 | 0.099 | - 0.748 | 0.065 |
| 2-step games | 0.850 | 0.195 | 4.36 | < 0.001 | 0.468 | 1.232 |
| 3-step games | 1.459 | 0.210 | 6.96 | < 0.001 | 1.049 | 1.870 |
| Baseline treatment |  |  |  |  |  |  |
| C-1-step condition |  |  |  |  |  |  |
| 1-step games | 0.051 | 0.320 | 0.16 | 0.873 | - 0.577 | 0.679 |
| 2-step games | - 0.922 | 0.276 | - 3.34 | 0.001 | - 1.462 | - 0.381 |
| 3-step games | - 0.489 | 0.353 | - 1.38 | 0.166 | - 1.181 | 0.203 |
| C-2-step condition |  |  |  |  |  |  |
| 1-step games | 0.216 | 0.380 | 0.57 | 0.570 | - 0.529 | 0.961 |
| 2-step games | 0.436 | 0.297 | 1.47 | 0.141 | - 0.145 | 1.018 |
| 3-step games | 0.513 | 0.387 | 1.32 | 0.185 | -0.246 | 1.272 |
| C-3-step condition |  |  |  |  |  |  |
| 1-step games | -0.000 | 0.345 | - 0.00 | 1.000 | - 0.646 | 0.423 |
| 2-step games | -0.111 | 0.273 | - 0.41 | 0.683 | - 0.562 | - 0.451 |
| 3-step games | 0.000 | 0.312 | 0.00 | 1.000 | - 0.612 | - 0.612 |
| N. obs | 6900 |  |  |  |  |  |
| N. groups | 230 |  |  |  |  |  |

**Table S3.** Simple effects of Model 1. We report, for each combination of treatment, condition, and game class, the effect of Phase (Reassessment - Assessment) on Nash choice.

**Model 2**

Simple effects: effect of CRT-level

| Nash choice  (High-CRT - Low-CRT) | B | Std. Err. | z | p | 95% Conf. Inter. | |
| --- | --- | --- | --- | --- | --- | --- |
| 1-step games | 0.291 | 0.168 | 1.73 | 0.083 | -0.038 | 0.620 |
| 2-step games | 0.745 | 0.154 | 4.85 | < 0.001 | 0.444 | 1.046 |
| 3-step games | -0.376 | 0.171 | -2.19 | 0.028 | -0.711 | -0.040 |
| N. obs | 3450 |  |  |  |  |  |
| N. groups | 230 |  |  |  |  |  |

**Table S4.** Simple effects of Model 3. We report, for game class, the effect of the CRT group (High-CRT - Low-CRT) in the Assessment phase, across all treatments and conditions.

**Model 3**

Simple effects: effect of WM-level

| Nash choice  (High-WM - Low-WM) | B | Std. Err. | z | p | 95% Conf. Inter. | |
| --- | --- | --- | --- | --- | --- | --- |
| 1-step games | 0.442 | 0.168 | 2.62 | 0.009 | 0.112 | 0.772 |
| 2-step games | 0.326 | 0.152 | 2.14 | 0.032 | 0.028 | 0.625 |
| 3-step games | -0.541 | 0.173 | -3.13 | 0.002 | -0.879 | -0.202 |
| N. obs | 3450 |  |  |  |  |  |
| N. groups | 230 |  |  |  |  |  |

**Table S5.** Simple effects of Model 3. We report, for game class, the effect of the WM group (High-WM - Low-WM) in the Assessment phase, across all treatments and conditions.

**Model 4**

Omnibus results:

| Nash choice | df | chi2 | p |
| --- | --- | --- | --- |
| CRT group | 1 | 22.15 | < 0.001 |
| Condition | 2 | 2.74 | 0.254 |
| CRT group*Condition | 2 | 6.12 | 0.047 |
| Game class | 2 | 545.32 | < 0.001 |
| CRT group*Game class | 2 | 21.92 | < 0.001 |
| Condition*Game class | 4 | 54.48 | < 0.001 |
| CRT group*Condition*Game class | 4 | 25.40 | < 0.001 |
| Phase | 1 | 33.53 | < 0.001 |
| CRT group*Phase | 1 | 20.26 | < 0.001 |
| Condition*Phase | 2 | 11.61 | 0.003 |
| CRT group*Condition*Phase | 2 | 11.10 | 0.004 |
| Game class*Phase | 2 | 29.51 | < 0.001 |
| CRT group*Game class*Phase | 2 | 8.72 | 0.013 |
| Condition*Game class*Phase | 4 | 59.19 | < 0.001 |
| CRT group*Condition*Game class*Phase | 4 | 35.64 | < 0.001 |
| N. obs | 4770 |  |  |
| N. groups | 159 |  |  |

**Table S6**. Omnibus results of Model 4.

Simple effects: effect of CRT group*Phase

| Nash choice  CRT group (High -Low)*  Phase (Reass.-Assess.) | B | Std. Err. | z | p | 95% Conf. Inter. | |
| --- | --- | --- | --- | --- | --- | --- |
| C-1-step condition |  |  |  |  |  |  |
| 1-step games | 1.104 | 0.416 | 2.65 | 0.008 | 0.289 | 1.919 |
| 2-step games | 0.482 | 0.378 | 1.28 | 0.202 | - 0.258 | 1.223 |
| 3-step games | 1.964 | 0.407 | 4.82 | < 0.001 | 0.165 | 2.762 |
| C-2-step condition |  |  |  |  |  |  |
| 1-step games | 0.605 | 0.417 | 1.45 | 0.147 | - 0.212 | 1.422 |
| 2-step games | 0.601 | 0.419 | 1.43 | 0.152 | - 0.220 | 1.422 |
| 3-step games | - 1.057 | 0.460 | - 2.30 | 0.022 | - 1.196 | - 0.156 |
| C-3-step condition |  |  |  |  |  |  |
| 1-step games | 0.124 | 0.440 | 0.28 | 0.777 | - 0.737 | 0.986 |
| 2-step games | - 0.656 | 0.459 | - 1.43 | 0.153 | - 1.555 | 0.243 |
| 3-step games | 2.647 | 0.466 | 5.68 | < 0.001 | 1.734 | 3.561 |
| N. obs | 4770 |  |  |  |  |  |
| N. groups | 159 |  |  |  |  |  |

**Table S7.** Simple effects of Model 4. We report, for each combination of condition and game class, the interaction between the effect of the CRT group (High-CRT-Low-CRT) and the effect of Phase (Reassessment - Assessment) on Nash choice in the Feedback treatment.

**Model 5**

Omnibus results:

| Nash choice | df | chi2 | p |
| --- | --- | --- | --- |
| Nback group | 1 | 2.41 | 0.121 |
| Condition | 2 | 0.32 | 0.854 |
| Nback group*Condition | 2 | 0.65 | 0.723 |
| Game class | 2 | 585.81 | < 0.001 |
| Nback group*Game class | 2 | 23.40 | < 0.001 |
| Condition*Game class | 4 | 60.25 | < 0.001 |
| Nback group*Condition*Game class | 4 | 4.11 | 0.391 |
| Phase | 1 | 26.05 | < 0.001 |
| Nback group*Phase | 1 | 0.12 | 0.729 |
| Condition*Phase | 2 | 9.92 | 0.007 |
| Nback group*Condition*Phase | 2 | 0.31 | 0.855 |
| Game class*Phase | 2 | 28.63 | < 0.001 |
| Nback group*Game class*Phase | 2 | 11.45 | 0.003 |
| Condition*Game class*Phase | 4 | 51.09 | < 0.001 |
| Nback group*Condition*Game class*Phase | 4 | 9.51 | 0.050 |
| N. obs | 4770 |  |  |
| N. groups | 159 |  |  |

**Table S8**. Omnibus results of Model 5.

**Model 4b**

Omnibus results:

| Nash choice | df | chi2 | p |
| --- | --- | --- | --- |
| CRT score | 1 | 34.69 | < 0.001 |
| Condition | 2 | 1.50 | 0.473 |
| CRT score*Condition | 2 | 7.17 | 0.028 |
| Game class | 2 | 553.98 | < 0.001 |
| CRT score*Game class | 2 | 22.84 | < 0.001 |
| Condition*Game class | 4 | 52.36 | < 0.001 |
| CRT score*Condition*Game class | 4 | 10.95 | 0.027 |
| Phase | 1 | 29.84 | < 0.001 |
| CRT score*Phase | 1 | 17.92 | < 0.001 |
| Condition*Phase | 2 | 10.72 | 0.005 |
| CRT score*Condition*Phase | 2 | 4.49 | 0.106 |
| Game class*Phase | 2 | 30.39 | < 0.001 |
| CRT score*Game class*Phase | 2 | 6.39 | 0.041 |
| Condition*Game class*Phase | 4 | 51.23 | < 0.001 |
| CRT score*Condition*Game class*Phase | 4 | 37.29 | < 0.001 |
| N. obs | 4770 |  |  |
| N. groups | 159 |  |  |

**Table S9**. Omnibus results of Model 4b.

**Model 5b.**

Omnibus results:

| Nash choice | df | chi2 | p |
| --- | --- | --- | --- |
| N-Back score | 1 | 4.72 | 0.030 |
| Condition | 2 | 0.65 | 0.723 |
| N-Back score*Condition | 2 | 0.04 | 0.981 |
| Game class | 2 | 585.24 | < 0.001 |
| N-Back score*Game class | 2 | 18.63 | < 0.001 |
| Condition*Game class | 4 | 53.62 | < 0.001 |
| N-Back score*Condition*Game class | 4 | 8.78 | 0.067 |
| Phase | 1 | 27.48 | < 0.001 |
| N-Back score*Phase | 1 | 3.75 | 0.053 |
| Condition*Phase | 2 | 11.50 | 0.003 |
| N-Back score*Condition*Phase | 2 | 0.70 | 0.706 |
| Game class*Phase | 2 | 29.05 | < 0.001 |
| N-Back score*Game class*Phase | 2 | 3.70 | 0.157 |
| Condition*Game class*Phase | 4 | 54.66 | < 0.001 |
| N-Back score*Condition*Game class*Phase | 4 | 10.63 | 0.031 |
| N. obs | 4770 |  |  |
| N. groups | 159 |  |  |

**Table S10**. Omnibus results of Model 4b.

**Process tracing**

| **Treatment** | **Condition** | **IAS** | **Assessment** | **Reassessment** |
| --- | --- | --- | --- | --- |
| Low-CRT | C-1-Step | IAS-1-step | 0.28 (0.38) | 0.29 (0.42) |
|  |  | IAS-2-step | 0.17 (0.29) | 0.08 (0.20) |
|  |  | IAS-3-step | 0.17 (0.28) | 0.18 (0.32) |
|  | C-2-step | IAS-1-step | 0.17 (0.31) | 0.10 (0.28) |
|  |  | IAS-2-step | 0.30 (0.39) | 0.42 (0.49) |
|  |  | IAS-3-step | 0.07 (0.16) | 0.02 (0.07) |
|  | C-3-step | IAS-1-step | 0.30 (0.37) | 0.28 (0.42) |
|  |  | IAS-2-step | 0.09 (0.18) | 0.28 (0.42) |
|  |  | IAS-3-step | 0.18 (0.24) | 0.18 (0.34) |
| High-CRT | C-1-Step | IAS-1-step | 0.18 (0.29) | 0.51 (0.48) |
|  |  | IAS-2-step | 0.45 (0.43) | 0.22 (0.39) |
|  |  | IAS-3-step | 0.10 (0.20) | 0.48 (0.48) |
|  | C-2-step | IAS-1-step | 0.43 (0.40) | 0.07 (0.21) |
|  |  | IAS-2-step | 0.30 (0.37) | 0.70 (0.43) |
|  |  | IAS-3-step | 0.31 (0.34) | 0.02 (0.07) |
|  | C-3-step | IAS-1-step | 0.10 (0.20) | 0.48 (0.49) |
|  |  | IAS-2-step | 0.63 (0.41) | 0.29 (0.43) |
|  |  | IAS-3-step | 0.09 (0.19) | 0.47 (0.48) |

**Table S11.** Descriptive statistics reporting the average proportion of completion of each IAS (IAS-1-step; IAS-2-step; IAS-3-step) by condition (C-1-step; C-2-step; C-3-step) across all game classes in the Feedback treatment. Between-subject standard deviations in brackets.

**Model 6**

Omnibus results:

| IAS-1-step | df | chi2 | p |
| --- | --- | --- | --- |
| CRT group | 1 | 0.43 | 0.514 |
| Condition | 2 | 7.12 | 0.028 |
| CRT group*Condition | 2 | 2.60 | 0.272 |
| Game class | 2 | 0.84 | 0.658 |
| CRT group*Game class | 2 | 4.05 | 0.132 |
| Condition*Game class | 4 | 1.42 | 0.841 |
| CRT group*Condition*Game class | 4 | 1.64 | 0.801 |
| Phase | 1 | 1.81 | 0.179 |
| CRT group*Phase | 1 | 25.57 | < 0.001 |
| Condition*Phase | 2 | 228.34 | < 0.001 |
| CRT group*Condition*Phase | 2 | 121.02 | < 0.001 |
| Game class*Phase | 2 | 0.49 | 0.784 |
| CRT group*Game class*Phase | 2 | 0.17 | 0.918 |
| Condition*Game class*Phase | 4 | 1.25 | 0.870 |
| CRT group*Condition*Game class*Phase | 4 | 4.41 | 0.354 |
| N. obs | 4770 |  |  |
| N. groups | 159 |  |  |

**Table S12**. Omnibus results of Model 6.

Simple effects: effect of CRT group*Phase

| IAS-1-step  CRT group (High -Low)*  Phase (Reass.-Assess.) | B | Std. Err. | z | p | 95% Conf. Inter. | |
| --- | --- | --- | --- | --- | --- | --- |
| C-1-step condition |  |  |  |  |  |  |
| 1-step games | 2.82 | 0.588 | 4.80 | < 0.001 | 1.667 | 3.970 |
| 2-step games | 2.79 | 0.583 | 4.78 | < 0.001 | 1.643 | 3.929 |
| 3-step games | 2.88 | 0.583 | 4.94 | < 0.001 | 1.737 | 4.023 |
| C-2-step condition |  |  |  |  |  |  |
| 1-step games | - 2.64 | 0.823 | - 3.20 | < 0.001 | - 4.249 | - 1.023 |
| 2-step games | - 2.79 | 0.814 | - 3.43 | < 0.001 | - 4.386 | - 1.197 |
| 3-step games | - 4.10 | 0.944 | - 4.35 | < 0.001 | - 5.953 | - 2.252 |
| C-3-step condition |  |  |  |  |  |  |
| 1-step games | 4.15 | 0.710 | 5.84 | < 0.001 | 2.758 | 5.541 |
| 2-step games | 3.66 | 0.668 | 5.48 | < 0.001 | 2.353 | 4.972 |
| 3-step games | 5.26 | 0.744 | 7.07 | < 0.001 | 3.804 | 6.719 |
| N. obs | 4770 |  |  |  |  |  |
| N. groups | 159 |  |  |  |  |  |

**Table S13.** Simple effects of Model 6. We report, for each combination of condition and game class, the interaction between the effect of the CRT group (High-CRT-Low-CRT) and the effect of Phase (Reassessment - Assessment) on IAS-1-step in the Feedback treatment.

**Model 7**

Omnibus results:

| IAS-2-step | df | chi2 | p |
| --- | --- | --- | --- |
| CRT group | 1 | 26.71 | < 0.001 |
| Condition | 2 | 11.19 | 0.004 |
| CRT group*Condition | 2 | 2.54 | 0.281 |
| Game class | 2 | 0.62 | 0.735 |
| CRT group*Game class | 2 | 2.35 | 0.309 |
| Condition*Game class | 4 | 5.47 | 0.242 |
| CRT group*Condition*Game class | 4 | 0.40 | 0.982 |
| Phase | 1 | 8.22 | 0.004 |
| CRT group*Phase | 1 | 26.86 | < 0.001 |
| Condition*Phase | 2 | 245.74 | < 0.001 |
| CRT group*Condition*Phase | 2 | 183.90 | < 0.001 |
| Game class*Phase | 2 | 0.77 | 0.680 |
| CRT group*Game class*Phase | 2 | 2.72 | 0.257 |
| Condition*Game class*Phase | 4 | 1.38 | 0.848 |
| CRT group*Condition*Game class*Phase | 4 | 1.72 | 0.786 |
| N. obs | 4770 |  |  |
| N. groups | 159 |  |  |

**Table S14**. Omnibus results of Model 7.

Simple effects: effect of CRT group*Phase

| IAS-2-step  CRT group (High -Low)*  Phase (Reass.-Assess.) | B | Std. Err. | z | p | 95% Conf. Inter. | |
| --- | --- | --- | --- | --- | --- | --- |
| C-1-step condition |  |  |  |  |  |  |
| 1-step games | - 0.74 | 0.603 | - 1.23 | 0.219 | - 1.924 | 0.441 |
| 2-step games | - 0.35 | 0.592 | - 0.59 | 0.555 | - 1.510 | 0.810 |
| 3-step games | 0.19 | 0.648 | 0.30 | 0.766 | - 1.078 | 1.463 |
| C-2-step condition |  |  |  |  |  |  |
| 1-step games | 1.77 | 0.702 | 2.53 | 0.011 | 0.400 | 3.150 |
| 2-step games | 2.70 | 0.704 | 3.84 | < 0.001 | 1.320 | 4.080 |
| 3-step games | 2.96 | 0.723 | 4.10 | < 0.001 | 1.545 | 4.380 |
| C-3-step condition |  |  |  |  |  |  |
| 1-step games | - 6.34 | 0.717 | - 8.85 | < 0.001 | - 7.750 | - 4.939 |
| 2-step games | - 5.34 | 0.678 | - 7.87 | < 0.001 | - 6.670 | - 4.011 |
| 3-step games | - 6.29 | 0.718 | - 8.77 | < 0.001 | - 7.698 | - 4.885 |
| N. obs | 4770 |  |  |  |  |  |
| N. groups | 159 |  |  |  |  |  |

**Table S15.** Simple effects of Model 7. We report, for each combination of condition and game class, the interaction between the effect of the CRT group (High-CRT-Low-CRT) and the effect of Phase (Reassessment - Assessment) on IAS-2-step in the Feedback treatment.

**Model 8**

Omnibus results:

| IAS-3-step | df | chi2 | p |
| --- | --- | --- | --- |
| CRT group | 1 | 2.89 | 0.089 |
| Condition | 2 | 13.14 | 0.001 |
| CRT group*Condition | 2 | 0.76 | 0.684 |
| Game class | 2 | 5.77 | 0.056 |
| CRT group*Game class | 2 | 6.04 | 0.049 |
| Condition*Game class | 4 | 0.71 | 0.950 |
| CRT group*Condition*Game class | 4 | 4.65 | 0.325 |
| Phase | 1 | 3.88 | 0.049 |
| CRT group*Phase | 1 | 25.23 | < 0.001 |
| Condition*Phase | 2 | 163.99 | < 0.001 |
| CRT group*Condition*Phase | 2 | 68.12 | < 0.001 |
| Game class*Phase | 2 | 0.13 | 0.936 |
| CRT group*Game class*Phase | 2 | 0.71 | 0.700 |
| Condition*Game class*Phase | 4 | 0.78 | 0.942 |
| CRT group*Condition*Game class*Phase | 4 | 8.12 | 0.087 |
| N. obs | 4770 |  |  |
| N. groups | 159 |  |  |

**Table S16**. Omnibus results of Model 8.

Simple effects: effect of CRT group*Phase

| IAS-3-step  CRT group (High -Low)*  Phase (Reass.-Assess.) | B | Std. Err. | z | p | 95% Conf. Inter. | |
| --- | --- | --- | --- | --- | --- | --- |
| C-1-step condition |  |  |  |  |  |  |
| 1-step games | 3.63 | 0.658 | 5.51 | < 0.001 | 2.338 | 4.919 |
| 2-step games | 2.90 | 0.612 | 4.74 | < 0.001 | 1.705 | 4.105 |
| 3-step games | 3.60 | 0.623 | 5.79 | < 0.001 | 2.383 | 4.825 |
| C-2-step condition |  |  |  |  |  |  |
| 1-step games | - 2.55 | 1.187 | - 2.15 | - 0.032 | - 4.879 | - 0.225 |
| 2-step games | - 1.09 | 1.250 | - 0.88 | 0.381 | - 3.544 | 1.354 |
| 3-step games | - 4.80 | 1.289 | - 3.73 | < 0.001 | - 7.327 | - 2.275 |
| C-3-step condition |  |  |  |  |  |  |
| 1-step games | 4.69 | 0.785 | 5.97 | < 0.001 | 3.150 | 6.228 |
| 2-step games | 3.12 | 0.686 | 4.55 | < 0.001 | 1.778 | 4.466 |
| 3-step games | 5.13 | 0.777 | 6.60 | < 0.001 | 3.604 | 6.648 |
| N. obs | 4770 |  |  |  |  |  |
| N. groups | 159 |  |  |  |  |  |

**Table S17.** Simple effects of Model 6. We report, for each combination of condition and game class, the interaction between the effect of the CRT group (High-CRT-Low-CRT) and the effect of Phase (Reassessment - Assessment) on IAS-3-step in the Feedback treatment.

**Results of the entire sample (including participants who do not meet inclusion criteria)**

Descriptive statistics (Feedback vs. Baseline treatment, across conditions and games)

| **Treatment** | **Condition** | **Game class** | **Assessment** | **Reassessment** |
| --- | --- | --- | --- | --- |
| Feedback | C-1-Step | 1-step | 0.72 (0.33) | 0.69 (0.29) |
|  |  | 2-step | 0.56 (0.38) | 0.52 (0.37) |
|  |  | 3-step | 0.26 (0.27) | 0.44 (0.32) |
|  | C-2-step | 1-step | 0.70 (0.29) | 0.69 (0.32) |
|  |  | 2-step | 0.61 (0.33) | 0.78 (0.34) |
|  |  | 3-step | 0.24 (0.21) | 0.18 (0.23) |
|  | C-3-step | 1-step | 0.74 (0.29) | 0.68 (0.35) |
|  |  | 2-step | 0.50 (0.36) | 0.67 (0.37) |
|  |  | 3-step | 0.23 (0.27) | 0.49 (0.39) |
|  | All | All | 0.51 (0.17) | 0.57 (0.22) |
| Baseline | C-1-Step | 1-step | 0.76 (0.23) | 0.78 (0.24) |
|  |  | 2-step | 0.59 (0.33) | 0.38 (0.36) |
|  |  | 3-step | 0.20 (0.15) | 0.14 (0.21) |
|  | C-2-step | 1-step | 0.82 (0.18) | 0.85 (0.17) |
|  |  | 2-step | 0.59 (0.34) | 0.66 (0.36) |
|  |  | 3-step | 0.13 (0.17) | 0.19 (0.23) |
|  | C-3-step | 1-step | 0.80 (0.24) | 0.82 (0.18) |
|  |  | 2-step | 0.55 (0.38) | 0.53 (0.36) |
|  |  | 3-step | 0.26 (0.18) | 0.24 (0.24) |
|  | All | All | 0.52 (0.16) | 0.51 (0.15) |

**Table S18.** Descriptive statistics of the Feedback and Baseline treatment including the entire sample of participants (i.e., including participants not meeting the criteria of inclusion: see the “Data exclusion” section). The table reports the average proportion of Nash equilibrium choices (between-subject standard deviations in brackets) condition (C-1-step; C-2-step; C-3-step), game class (1-step; 2-step; 3-step) and phase (Reassessment; Assessment).

Descriptive statistics (Low-CRT vs. High-CRT, across conditions and games, in the Feedback treatment)

| **CRT level** | **Condition** | **Game class** | **Assessment** | **Reassessment** |
| --- | --- | --- | --- | --- |
| Low | C-1-Step | 1-Step | 0.72 (0.32) | 0.60 (0.28) |
|  |  | 2-step | 0.50 (0.38) | 0.41 (0.36) |
|  |  | 3-step | 0.29 (0.29) | 0.33 (0.24) |
|  | C-2-step | 1-Step | 0.63 (0.29) | 0.57 (0.34) |
|  |  | 2-step | 0.60 (0.31) | 0.71 (0.35) |
|  |  | 3-step | 0.24 (0.22) | 0.24 (0.25) |
|  | C-3-step | 1-Step | 0.72 (0.28) | 0.65 (0.35) |
|  |  | 2-step | 0.36 (0.29) | 0.59 (0.38) |
|  |  | 3-step | 0.25 (0.24) | 0.35 (0.35) |
|  | All | All | 0.48 (0.15) | 0.49 (0.19) |
| High | C-1-Step | 1-Step | 0.72 (0.35) | 0.81 (0.27) |
|  |  | 2-step | 0.65 (0.37) | 0.67 (0.33) |
|  |  | 3-step | 0.21 (0.24) | 0.59 (0.35) |
|  | C-2-step | 1-Step | 0.76 (0.29) | 0.79 (0.27) |
|  |  | 2-step | 0.61 (0.36) | 0.84 (0.32) |
|  |  | 3-step | 0.25 (0.20) | 0.12 (0.20) |
|  | C-3-step | 1-Step | 0.77 (0.29) | 0.72 (0.36) |
|  |  | 2-step | 0.73 (0.34) | 0.78 (0.34) |
|  |  | 3-step | 0.20 (0.31) | 0.70 (0.35) |
|  | All | All | 0.54 (0.18) | 0.66 (0.23) |

**Table S19.** Descriptive statistics of the Feedback treatment including the entire sample of participants (i.e., including participants not meeting the criteria of inclusion: see the “Data exclusion” section). The table reports the average proportion of Nash equilibrium choices (between-subject standard deviations in brackets) by CRT level (Low; High), condition (C-1-step; C-2-step; C-3-step), game class (1-step; 2-step; 3-step) and phase (Reassessment; Assessment).
